# Revisiting Fused‐Pyrrolo‐1,10‐Phenanthroline Derivatives: Novel Transformations and Stability Studies

**DOI:** 10.1002/open.202400365

**Published:** 2025-05-06

**Authors:** Cristina M. Al Matarneh, Alina Nicolescu, Sergiu Shova, Mircea Apostu, Razvan Puf, Francesca Mocci, Aatto Laaksonen, Ionel I. Mangalagiu, Ramona Danac

**Affiliations:** ^1^ Center of Advanced Research in Bionanoconjugates and Biopolymers “Petru Poni” Institute of Macromolecular Chemistry of Romanian Academy 41A Grigore Ghica Voda Alley Iasi 700487 Romania; ^2^ NMR Laboratory “Petru Poni” Institute of Macromolecular Chemistry of Romanian Academy 41A Grigore Ghica Voda Alley Iasi 700487 Romania; ^3^ Department of Inorganic Polymers “Petru Poni” Institute of Macromolecular Chemistry of Romanian Academy 41A Grigore Ghica Voda Alley Iasi 700487 Romania; ^4^ Faculty of Chemistry “Alexandru Ioan Cuza” University of Iasi 11 Carol I Iasi 700506 Romania; ^5^ Department of Chemical and Geological Sciences University of Cagliari Cagliari 09124 Italy; ^6^ Department of Materials and Environmental Chemistry Division of Physical ChemistryArrhenius Laboratory Stockholm University Stockholm 106 91 Sweden; ^7^ State Key Laboratory of Materials‐Oriented and Chemical Engineering Nanjing Tech University Nanjing 210009 P. R. China; ^8^ Department of Engineering Sciences and Mathematics Division of Energy Science Luleå University of Technology Luleå 97187 Sweden

**Keywords:** 1,10‐phenanthroline, conformational studies, DFT calculations, NMR spectroscopy

## Abstract

In this study, new pyrrolo[3',4':3,4]pyrrolo[1,2‐*a*][1,10]phenanthroline derivatives are developed and their stabilities and transformation pathways are investigated. The synthetic approach toward these novel derivatives include a pivotal [3 + 2] cycloaddition of in situ generated ylides, followed by cycloadducts oxidation and other unexpected transformations. The structures of the intermediate and final compounds are proposed based on information obtained from several spectral techniques. Stability study reveal that electron‐donating groups in the para position of the phenyl ring promote easier oxidation, whereas electron‐withdrawing substituents enhance the stability of the compounds. The acid–base titration of α‐monosubstituted 1,10‐phenanthroline **6a** results in a reversible color change, which is preliminarily explored through spectral methods.

## Graphical abstract

The synthesis of new pyrrolo[3',4':3,4]pyrrolo[1,2‐a][1,10]phenanthroline derivatives is performed starting from [3+2] cycloaddition of in situ generated ylides, followed by cycloadducts oxidation and other transformations. The structures of the unexpected intermediates and final compounds are obtained from nuclear magnetic resonance and single‐crystal X‐ray data. The influence of the para‐substituent electronic effect upon the compounds stability is investigated.

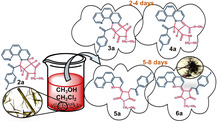

## 1 Introduction

1,10‐Phenanthroline, a bidentate ligand extensively used in coordination chemistry since its discovery in 1930s, has attracted considerable attention due to its structural versatility (hydrophobic, basic, aromatic, planar, and rigid) and numerous applications. Its distinct rigid structure, imposed by the central benzene with two fused pyridine rings that exhibit the chelate effect, entropically enables strong interaction with transition metal ions to produce stable complexes. Moreover, the extended conjugation over three fused aromatic cycles makes phenanthroline an electron‐deficient ligand, thus a strong π‐acceptor that tends to stabilize soft cations.^[^ ^1^, ^2^, ^3^, ^4^, ^5^ ^]^ Phenanthroline‐based derivatives and complexes have a variety of characteristics and applications ranging from building blocks in supramolecular architectures (i.e., rotaxanes, catenanes),^[^ ^6^, ^7^, ^8^, ^9^ ^]^ in luminescent molecules^[^ ^10^, ^11^, ^12^, ^13^, ^14^ ^]^ or catalysts,^[^ ^15^, ^16^, ^17^ ^]^ to the development of bioorganic reagents and probes tailored for biological and medical applications. Thus, some complexes possess interesting biological applications such as binding agent for proteins, RNA and DNA,^[^ ^18^, ^19^, ^20^ ^]^ stabilizers for DNA G‐quadruplexes^[^ ^21^, ^22^ ^]^ that are regarded as potential targets for anticancer drugs,^[^ ^23^, ^24^ ^]^ or metalloprotein inhibitors and DNA cleaving reagent, such as [Cu(phen)_2_]^2+^ complex, commonly used in molecular biology.^[^ ^25^ ^]^ Other phenanthrolines and substituted derivatives or metal complexes have been developed as potential drugs in treating different pathologies.^[^ ^26^, ^27^, ^28^, ^29^, ^30^, ^31^, ^32^, ^33^, ^34^, ^35^ ^]^

1,10‐Phenanthroline is also an essential chromophore in analytical chemistry being used to determine metal ions, especially iron, through spectrophotometric analysis.^[^ ^36^ ^]^

In this study, we revisited a previous investigation on the synthesis of compounds of type **2**,^[^ ^37^ ^]^ in which it was observed that these compounds alter their color upon exposure to air. However, at that time, the underlying process was not explored.

Given the vast range of domains in which 1,10‐phenanthroline has found applications, and as a continuation of our research on N‐heterocycle fused systems,^[^ ^38^, ^39^, ^40^, ^41^, ^42^ ^]^ we present herein an investigation regarding the transformation of type **2** [3 + 2] cycloadducts into α‐monosubstituted 1,10‐phenathroline derivatives and a preliminary study regarding the behavior of α‐monosubstituted 1,10‐phenathroline derivative **6a** under acid–base conditions.

## 2 Results and Discussion

### 2.1 Synthesis, Rearrangements, and Structural Characterization of Compounds 2a‐6a

**Scheme 1:**
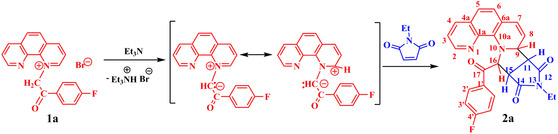
Synthesis of derivative **2a**.

**Figure 1:**
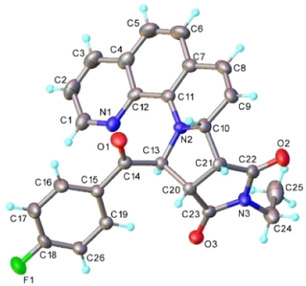
X‐ray molecular structure of **2a** with atom labeling and thermal ellipsoids at 40% level.

The synthesis of the α‐substituted phenanthroline **2a‐6a** is outlined in **Scheme** **1**. Compound **2a** was obtained in two steps following a method similar to that previously reported,^[^ ^32^, ^37^ ^]^ by reacting N‐ethylmaleimide (NEtMI) with in situ generated ylide of 1,10‐phenantrolinium monoquaternary salt **1a**. It is important to mention that basic conditions (Et_3_N) were employed to generate the intermediate ylides and compound **2a** was isolated through crystallization using methanol. Information from nuclear magnetic resonance (NMR) and infrared (IR) spectroscopies as well as single‐crystal X‐ray diffraction (XRD) (**Figure** **1**) confirmed the structure proposed for compound **2a**. ^1^H and ^13^C NMR spectra are presented in Figure S1 and S2, Supporting Information.

In the ^1^H NMR spectrum, the presence of para‐fluorobenzoyl group is straightforward confirmed from the specific signals shapes, owing to proton‐fluorine couplings: a triplet at 7.54 ppm (^3^ *J*_H,H_ = ^3^ *J*_H,F_ = 9 Hz) and a doublet of doublets at 8.26 ppm (^3^ *J*_H,H_ = 9 Hz, ^4^ *J*_H,F_ = 5 Hz). 1,10‐Phenantroline group has characteristic signals with the shapes defined mainly by first order proton‐proton scalar couplings. Three spin systems were assigned from the H,H‐COSY spectrum (Figure S3, Supporting Information): H‐2, H‐3, and H‐4 represented by three doublets of doublet at 7.18, 7.74, and 8.07 ppm, H‐5 and H‐6 with two doublets at 7.16 and 7.24 ppm and the third spin system containing H‐7, H‐8, and H‐9 phenanthroline protons (two doublets of doublet at 6.07 and 6.55 ppm, respectively, and a multiplet in the interval 5.53–5.55 ppm) along to the protons H‐11 and H‐15 of the tetrahydropyrrole ring. The unexpected singlet shape of proton H‐16 is the result of the dihedral angle between H‐16 and H‐15. As it can be seen from the X‐ray molecular structure of **2a** (Figure 1), this angle is close to 90°, thus the coupling constant is close to 0. The exact proton and carbon assignments are given in the supplementary material.

The structure of the compound **2a** was further confirmed by XRD (Figure 1) (crystals phase purity analyzed by powder X‐ray diffraction (PXRD) (Figure S5, Supporting Information)). The bond distances and angles are summarized in the Table S1, Supporting Information. The analysis of the packing diagram has evidenced that the adjacent molecules are interacting through C—H…O hydrogen bonds to form one‐dimensional arrays running along *b* axis, as shown in Figure S4, Supporting Information.

**Scheme 2:**
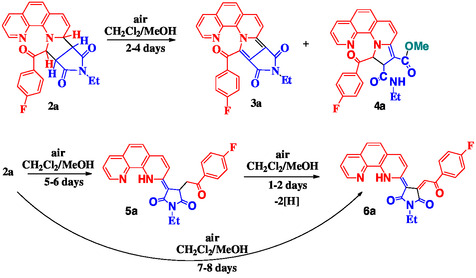
The pathways for obtaining derivatives **3a–6a**.

After several hours of exposure to air and light, compound **2a** changed its color from yellow to red, indicating increased environmental sensitivity, most likely a result of oxidation phenomenon. To investigate this peculiar behavior, we dissolved compound **2a** in a mixture of CH_2_Cl_2_/MeOH aiming to accelerate the process. The resulting mixture was then periodically investigated using column chromatography with the same solvent system (CH_2_Cl_2_/MeOH 98/2, v/v), at intervals of 1–2 days. No traces of the original compound **2a** were found; instead, we isolated the following new compounds: compound **3a** formed through aromatization of the tetrahydropyrrolo ring, appeared after 2 days, compound **4a** resulting from partial dehydrogenation of the tetrahydropyrrolo ring and the subsequent hydrolysis of the imide cycle was isolated after ≈3–4 days, compound **5a** that is an α‐monosubstituted 1,10‐phenanthroline derivative likely formed through an unusual opening of the newly formed pyrrolo cycle of **2a** was separated after 6–7 days, and compound **6a** was the last to be obtained after 7–8 days and remained stable no matter how long it was left in CH_2_Cl_2_/MeOH mixture (**Scheme** **2**). Following isolation, compound **5a** was also immersed in a CH_2_Cl_2_/MeOH mixture, which, after 1–2 days, resulted in the formation of compound **6a** through an oxidative dehydrogenation process (Scheme 2).

The isolated compounds **3a–6a** were analyzed through NMR spectroscopy. Thus, in the proton and carbon spectra of compound **3a** (Figure S6 and S7, Supporting Information), there are no signals in the aliphatic region, except those assigned to N‐ethyl group. The signals from the aromatic region are similar with those corresponding to 1,10‐phenantroline and para‐fluorobenzoyl groups, previously identified in compound **2a**, indicating that these groups are unaffected in **3a**. Four new carbon signals were identified in **3a**, at 110.9 (C‐11), 126.6 (C‐15), 128.4 (C‐16), and 131.3 (C‐9) ppm, in accordance with the proposed aromatization of the tetrahydropyrrolo ring from compound **2a**. Compound **4a** has a distinctive NMR spectral fingerprint, with characteristic signals for newly formed functional groups (Figure S8 and S9, Supporting Information). The singlet from 3.80 ppm assigned to methyl protons and carboxyl signal from 168.1 ppm indicate the presence of carboxymethyl group. The signals for N‐ethyl amide group are similar with those previously identified in compounds **2a** and **3a** for N‐ethyl‐succinimide group (at 1.14 and 3.31 ppm for ethyl protons and 171.1 ppm for amidic carbonyl). The formation of dihydropyrrole ring is sustained by two new doublets at 3.98 (H‐12) and 8.32 (H‐13) ppm, the last one being highly deshielded by both nitrogen and oxygen. Directly linked carbons resonate at 49.3 (C‐12) and 69.7 (C‐13) ppm.

For compound **5a**, we identify several proton and carbon signals that support the structural particularities (Figure S10 and S11, Supporting Information). In the proton spectrum, the amine proton resonates at 13.49 ppm, highly deshielded due to hydrogen bond formed with succinimide oxygen. The methylene protons have a specific diastereotopic pattern caused by the chiral CH, each proton giving rise to a doublet of doublets centered at 3.51 and 4.00 ppm. For the CH coupling partner, we identified a doublet of doublets centered at 3.88 ppm. In the carbon spectrum, the exocyclic double bond has two signals at 87.8 ppm (C‐11) and 143.8 ppm (C‐9), the last one, covalently linked to nitrogen, resonating at higher frequency due to nitrogen electron‐withdrawing effect.

**Figure 2:**
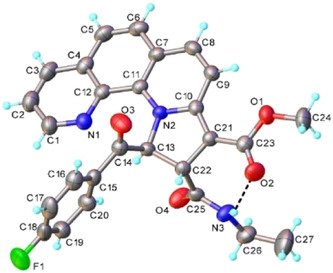
X‐ray molecular structure of **4a** with atom labeling and thermal ellipsoids at 50% level. Hydrogen bond parameters: N3—H…O2 [N3‐H 0.86 Å, H…O2 2.15 Å, N3…O2 2.831(2) Å, ∠N3HO2 136.0°.

**Figure 3:**
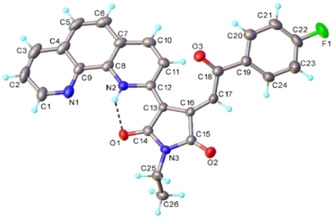
X‐ray molecular structure of **5a** with atom labeling and thermal ellipsoids at 50% level. Hydrogen bond parameters: N2—H…O1 [N2—H 0.88 Å, H…O1 1.93 Å, N2…O1 2.657(2) Å, ∠N2HO1 138.4°.

The last compound in this series of transformations, compound **6a**, had an NMR pattern similar with **5a**, indicating few structural differences between the two structures (Figure S12 and S13, Supporting Information). The main difference was the absence of proton aliphatic signals from interval 3.70 to 4.10 ppm and the appearance of a new singlet at 7.28 ppm. Based on the information obtained from bidimensional proton‐carbon direct and long‐range correlation experiments, we proposed the structure for **6a**, as indicated in Scheme 2. Thus, from H,C‐HSQC experiment (Figure S14a, Supporting Information) we obtained the chemical shift value for the directly linked carbon at 106.4 ppm, specific to unsaturated CH group. In the long‐range H,C‐HMBC experiment (Figure S14b, Supporting Information) we obtained correlation signals between this proton and carbonyl from succinimide (169.9 ppm) and ketone group (186 ppm). The exact proton and carbon assignments are given in the supplementary material suitable crystals for XRD were obtained and used to authenticate the structures of compounds **4a** (**Figure** **2**, Table S1, Supporting Information) and **5a** (**Figure** **3**, Table S1, Supporting Information).

In the case of compound **4a**, it is worth mentioning the intramolecular hydrogen bond where amino group acts as donor towards carboxylate oxygen atom. Further analysis of the crystal structure has revealed the presence of a two‐dimensional network stabilized by C—H…F and C—H…O—H bonds and quite strong *π–π* stacking interactions (Figure S15, Supporting Information). The main crystal structure motif results from the packing of the discrete 2D layers in parallel orientation to *ab* plane (Figure S15, Supporting Information).

Compound **5a** exhibits a molecular crystal structure with one neutral unit in the asymmetric part of the unit cell.

As it can be seen from the packing drawing (Figure S16, Supporting Information), adjacent molecules are involved in intermolecular interactions through weak C—H…O2 hydrogen bonds and stacking interactions between 1,10‐phenanthroline moieties, resulting a three‐dimensional architecture.

Similar with compound **2a**, the experimental PXRD pattern obtained for derivative **5a** aligns well with the simulated one, confirming **5a's** crystals phase purity (Figure S17, Supporting Information). Unfortunately, for compound **4a**, we were unable to obtain a powder XRD pattern due to its poor stability in air.

The transformation of compound **2a** into compound **6a** was also verified through ESI‐MS spectrometry (positive mode) (Figure S18, Supporting Information). For compound **2a** we detected the signal corresponding to *m*/*z* 442.1633 as single charge protonated ion [M + H]^+^, while the molecular ion signal for **6a** appeared at *m*/*z* 440.1560, confirming the proposed structures.

Given that **6a** exhibits a conjugated system with a donor–acceptor topology, we were particularly interested in exploring the photophysical properties of phenanthroline within this framework.

**Figure 4:**
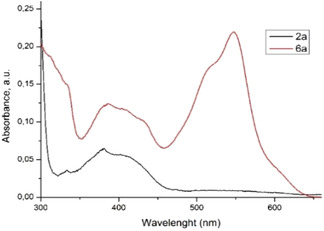
UV–Vis absorption spectra in DMSO (20 μm) of phenanthroline compounds **2a** (black) and **6a** (red).

For this, UV‐Vis absorption spectrum of **6a** was measured in dimethylsulfoxide (DMSO) Fourier transform infrared and compared to that of the starting compound **2a**. According to **Figure** **4**, **6a** exhibited a spectral profile with a three‐band system, having the main maxima centered at ≈330, 380, and 540 nm. Since the two low wavelength absorption bands (from the UV domain) perfectly overlap with those registered for **2a**, they can be accurately assigned to the *π*–π* transitions and *n *→ π* transitions occurring in the 1,10‐phenanthrolin‐2(1*H*)‐ylidene system.

### 2.2 Conformational Study

A conformational study was conducted on compound **5a** in order to comprehend the structures present in the proposed transformational scheme. The primary parameter under investigation was the position of the para‐fluorophenyl group in relation to the phenanthroline group.

**Figure 5:**
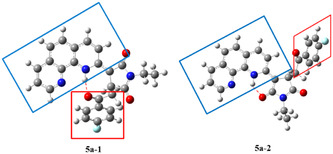
The two stable conformers of **5a**: **5a‐1** with the two groups of interest on the same side and **5a‐2** with the two groups on opposite sides. Color code: gray (carbon), white (hydrogen), blue (nitrogen), red (oxygen), and cyan (fluorine).

Understanding the favorable position is crucial for predicting the step in which the molecule of interest undergoes rearrangement. Two potential spatial arrangement of the fluorophenyl group were analyzed (**Figure** **5**). In one arrangement (**5a‐1**), the group is positioned on the same side as the phenanthroline core, similar to compound **4a**, while in the other arrangement (**5a‐2**) the two groups are on opposite sides, resembling the structure of compound **6a**.

Following the density functional theory (DFT) calculations, the energies corresponding to both structures were obtained. The specific energy for structure **5a‐1** is −1492.2525 Hartree, while for structure **5a‐2** it is −1492.2473 Hartree. The energy difference (Δ*E*) between the two structures amounts to 3.263 Kcal mol^−1^, with structure **5a‐2** exhibiting the lower energy and thus being more favorable.

### 2.3 Extended Syntheses of Compounds Type 2 and Their Stability

After elucidating the structures resulted from compound **2a** rearrangements (Scheme 2), we decided to broaden our research and investigate other similar derivatives. This expansion aimed to observe whether there are stability differences for this class of compounds. Therefore, we synthesized compounds **2b‐d** having donor substituents on the para position of the phenyl (‐Cl, ‐Br, ‐OMe) and compound **2e** having withdrawing effect substituent (‐NO_2_). These syntheses were carried out in similar conditions as in Scheme 1 (CH_2_Cl_2_, Et_3_N, NEtMI, room temperature, 24 h) using the corresponding phenathrolin‐1‐ium monoquaternary salts. The resulted compounds showed proton spectral fingerprints similar to those previously described by us.^[^ ^37^ ^]^

For the stability study, compounds **2b‐d** have been kept several days in a CH_2_Cl_2_/MeOH 1/1v/v solution. The resulting mixtures were then subjected to column chromatography, and the pure fractions were analyzed using NMR spectroscopy.

**Scheme 3:**
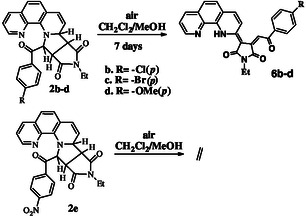
Synthesis of compounds **6b–d** starting from compounds type **2**.

Under the given experimental conditions, compound **2e** exhibited remarkable stability and did not undergo any detectable modifications. In contrast, the other three compounds (**2b–d**) experienced the same transformation as compound **2a**. However, unlike **2a**, for these compounds we were able to isolate and characterize only the final transformation products, designated as **6b–d**. This suggests that, while all four compounds shared a common reaction pathway, the stability and isolability of intermediates varied among them, with compound **2e** standing out due to its resistance to modification. (**Scheme** **3**).

The proton NMR spectra acquired for compounds **6b‐d** are included in Supporting Information (Figure S22–S25, Supporting Information). The spectral profiles are very similar with that obtained for compound **6a**, when it was analyzed in CDCl_3_ (see Figure S21, Supporting Information). The only significant differences were observed for the proton signals assigned to the para‐substituted phenyl residue.

Based on these findings, we concluded that the presence of a stronger electron‐donating substituent on the phenyl ring at the para position in compounds **2** facilitates their oxidation, making them more susceptible to different transformations. This observation suggests that increased electron density in the aromatic system promotes oxidation. Conversely, substituents with an electron‐withdrawing effect enhance the stability of these compounds by reducing their susceptibility to oxidation, likely due to a decrease in electron density on the aromatic ring. This correlation between substituent effects and oxidation behavior provides valuable insight into the electronic influence on the reactivity and stability of these compounds, which could be further explored for fine‐tuning their properties in future studies.

### 2.4 Preliminary pH Dependent Properties Investigation

**Figure 6:**
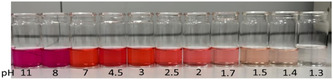
Color change of compound **6a** from basic (left) to acidic (right) conditions. The pH values are annotated on the Figure, the initial solution having pH = 7.

Compound **6a** was subjected to further investigation due to its intriguing behavior under acid–base conditions. When exposed to a basic medium, the color of the organic solution changes from ruby‐red (pH = 7) to purple (pH = 8, pH = 11), while in acidic conditions it becomes colorless (pH = 1.3) (**Figure** **6**). Notably, this color change is reversible, occurring both from basic to acidic and from acidic to basic conditions.

The compound stability in both media was evaluated through MS analysis. It was found that the exact mass associated with compound **6a** after acidic titration with HCl remained *m*/*z* = 440, indicating the presence of an isomer (Figure S19, Supporting Information). In basic media, after titration with NaOH, we found the signal at *m*/*z* = 462, that can be attributed to an [M‐H + Na]^+^ ion (Figure S20, Supporting Information).

**Figure 7:**
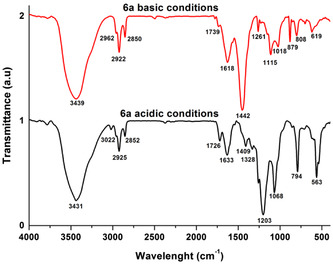
FT‐IR spectra of compound **6a** in acidic (black line) and basic media (red line).

The Fourier transform infrared (FTIR) spectra of compound **6a** in acidic and basic conditions show several differences (**Figure** **7**). In acidic media, an absorption band appears at 3022 cm^−^^1^, indicating the presence of a C—H bond, which is absent in basic conditions. Other bands in this region exhibit slight shifts, suggesting proton movement along the structure. A strong band at 1442 cm^−^^1^ is present in basic media, while an absorption at 1328 cm^−^^1^ is observed only in acidic conditions, possibly indicating the presence of a methylene group. This suggests that a CH‐to‐CH^2^ transformation occurs due to proton movement.

In the 750–1300 cm^−^^1^ range, three intense bands at 1261, 1203, and 1068 cm^−^^1^ are prominent in acidic media but shift and weaken in basic conditions. This supports the possibility of aromatization of the maleimide cycle, accompanied by a CH‐to‐CH^2^ transformation and potential aromatization of the phenanthroline core. Additionally, a band at 879 cm^−^^1^ is exclusive to basic media, possibly indicating the formation of a vinylidene structure absent in acidic conditions. Further differences appear in the 400–750 cm^−^^1^ range, where two intense bands at 563 and 538 cm^−^^1^ are observed in acidic media, while only a weaker band at 619 cm^−^^1^ is found in basic conditions.

**Figure 8:**
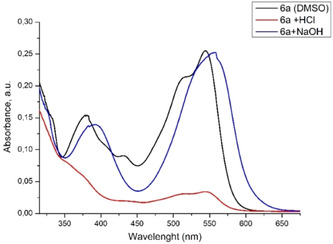
Comparative UV–Vis absorption spectra in DMSO of **6a** under neutral, acidic, and basic environments.

Going further with our investigations, we analyzed **6a** in both acidic and basic media by UV‐Vis spectroscopy (**Figure** **8**) and the optical responses were refereed to that of the neutral molecule. To this aim, the absorption spectra of **6a** was recorded in DMSO to which concentrated HCl or NaOH was added. At low pH values (1,2), all absorption bands significantly dropped in the intensity, which is contrary to the optical response at basic pH, when obvious bathochromic shifts of the absorption bands occurred.

## 3 Conclusion

In conclusion, we successfully synthesized and studied the stability of compounds **2a–e** with the 11a,12‐dihydro‐8aH‐pyrrolo[3',4':3,4]pyrrolo[1,2‐a][1,10]phenanthroline‐9,11(8bH,10H)‐dione structure. These derivatives were obtained through a [3 + 2] cycloaddition using 1,10‐phenanthrolin‐1‐ium monoquaternary salts and their in situ generated ylides. Stability studies revealed that electron‐donating groups in the para position of the phenyl ring promoted oxidation, as seen in compound **2a**, which underwent further ring opening to yield unexpected derivatives **5a** and **6a**. Electron‐withdrawing groups enhanced stability under air and CH_2_Cl_2_/MeOH conditions. The chemical structures of the new 1,10‐phenanthroline derivatives were confirmed by NMR, IR, MS, and XRD techniques. Notably, compound **6a** exhibited intriguing pH‐dependent behavior, suggesting potential for further exploration of its pH‐responsive properties and mechanisms.

## 4 Experimental Section

All the reagents and solvents employed were used without further purification.

Thin‐layer chromatography (TLC) was carried out on Merck silica gel 60F_254_ plates. Column chromatography was carried out on silica gel (Roth 60, 0.04–0.063 mm). Visualization of the plates was achieved using a UV lamp (*λ*_max_ = 254 or 365 nm).

Melting points were recorded on an A. Krüss Optronic Melting Point Meter KSPI and are uncorrected.

The NMR spectra included in this study were recorded on either Bruker Avance NEO 400 and 600 MHz or Bruker Avance III 500 MHz spectrometers equipped with 5 mm four nuclei direct detection z‐gradient probe (H,C,F,Si‐QNP) and 5 mm inverse detection multinuclear z‐gradient probes respectively. Proton and carbon chemical shifts are reported in *δ* units (ppm) relative to the residual solvent signal (ref: DMSO‐d_6_^1^H, 2.51 ppm and ^13^C, 39.47 ppm; CDCl_3_^1^H, 7.26 ppm and ^13^C, 77.01 ppm). H,H‐COSY (correlation spectroscopy), H,C‐HSQC (heteronuclear single quantum coherence) and H,C‐HMBC (heteronuclear multiple bond correlation) experiments were recorded using standard pulse sequences as delivered by Bruker with TopSpin 4.0.8 spectrometer control and processing software. The following abbreviations were used to designate chemical shift multiplicities: s = singlet, d = doublet, t = triplet, q = quartet, m = multiplet. Coupling constants are given in Hz.

IR spectra were recorded in transmission mode on a FTIR Shimadzu, Jasco 660 plus FTIR spectrophotometer or Bruker Vertex instrument, model 70.

MS data were acquired using an Agilent 6520 Series Accurate‐Mass Quadrupole Time‐of‐Flight (Q‐TOF) LC/MS instrument. The samples (solubilized in a mixture of water and acetonitrile) were injected into the electrospray ion source (ESI) via a syringe pump at a flow‐rate of 0.2 mL min^−1^. The running parameters of Q‐TOF MS were set as follows: electrospray ionization in positive ion mode; drying gas (N_2_) flow rate 7 L min^−1^; drying gas temperature 325 °C; nebulizer pressure 35 psig; capillary voltage 4000 V; fragmentation voltage 175 V; the full‐scan mass spectra of the examined compounds were acquired in the *m*/*z* range 100–1000. The mass scale was calibrated using the standard calibration procedure and standard compounds provided by the manufacturer. Data were collected and processed using MassHunter Workstation Software Data Acquisition for 6200/6500 Series, version B.07.00 (Agilent Technologies, Inc., Santa Clara, CA, USA).

Single crystal XRD data were collected on an Oxford‐Diffraction XCALIBUR Eos CCD diffractometer with graphite‐monochromated Mo‐Kα radiation. The unit cell determination and data integration were carried out using the CrysAlisPro package from Oxford Diffraction.^[^ ^43^ ^]^ The structure was solved with program SHELXT using the intrinsic phasing method and refined by the full‐matrix least‐squares method on *F* ^2^ with SHELXL.^[^ ^44^ ^]^ Olex2 was used as an interface to the SHELX programs.^[^ ^45^ ^]^ Non‐hydrogen atoms were refined anisotropically. Hydrogen atoms were added in idealized positions and refined using riding model. Selected crystallographic data and structure refinement details for are provided in **Table** **1**.

**Table 1:** Crystal data and details of structure refinement for 2a, 4a, and 5a.

Emp. formula	**2a** C_26_H_20_FN_3_O_3_	**4a** C_27_H_22_FN_3_O_4_	**5a** C_26_H_18_FN_3_O_3_
Fw	441.45	471.47	439.43
*T* [K]	293	293	180.00(14)
space group	*C*2/*c*	*P*‐1	*P*2_1_/*c*
*a* [Å]	30.341(4)	9.9451(6)	12.7869(9)
*b* [Å]	6.2903(8)	10.8062(8)	4.1473(2)
*c* [Å]	23.564(3)	11.2340(4)	38.298(3)
*α* [°]	90	96.719(4)	90
*β* [°]	110.418(17)	93.489(4)	93.695(6)
*γ* [°]	90	112.297(6)	90
*V* [Å^3^]	4214.7(11)	1102.07(12)	2026.7(2)
*Z*	8	2	4
*ρ*_calcd_ [g cm^−3^]	1.391	1.421	1.440
*μ* [mm^−1^]	0.099	0.102	0.102
Crystal size [mm]	0.30 × 0.10 × 0.10	0.30 × 0.20 × 0.05	0.35 × 0.03 × 0.03
2Θ range	3.688–50.05	4.122–58.892	3.722–50.054
Refls. collected	8309	12391	13578
Indep. Refls., *R*_int_	3694, 0.0498	5098, 0.0355	3544, 0.0581
Data/rests./params.	3694/0/299	5098/0/318	3544/0/300
GOF	1.086	1.036	1,017
*R*_1_	0.0889	0.0565	0.0523
*wR*_2_	0.1714	0.1436	0.1301
CCDC no.	2232740	2232739	2232741

PXRD analysis was performed on a Rigaku Miniflex 600 diffractometer using CuKα‐emission in the angular range 2°–50° (2*θ*) with a scanning step of 0.01° and a recording rate of 2° min^−1^.

UV‐Vis measurements were performed on a Lambda 35 device (Perkin Elmer, USA). The absorption spectra were measured in the 300–700 nm range for identical sample volumes (3 μL) with the following parameters: slit width 1 nm, scan speed 480 nm min^−1^ and data interval 1 nm. The spectra of the samples were measured at room temperature using 1 cm path length quartz cuvettes.

Computational experiment. To obtain optimized geometries of compounds **5a**, a DFT^[^ ^46^ ^]^ approach was employed with the B3LYP functional and 6‐311++G(d,p) basis set. In order to account for the solvent effect of dichloromethane (CH_2_Cl_2_) used during the experiment, the polarizable continuum model with integral equation formalism (IEFPCM)^[^ ^47^, ^48^ ^]^ was utilized. Gaussian 16 software package^[^ ^49^ ^]^ was employed for DFT calculations.

## Conflict of Interest

The authors declare no conflict of interest.

## Supporting information

Supplementary Material

## Data Availability

The data that support the findings of this study are available in the supplementary material of this article.
