# Supplementary material for: Revisiting Fused‐Pyrrolo‐1,10‐Phenanthroline Derivatives: Novel Transformations and Stability Studies
Source: ChemistryOpen. 2025 May 6;14(7):e202400365. doi: 10.1002/open.202400365 (PMC12256923; doi:10.1002/open.202400365)
Supplement: Supplementary file 1 — Supplementary Material [file OPEN-14-e202400365-s001.pdf]

Supplementary material for

Revisiting Fused Pyrrolo-1,10-Phenanthroline Derivatives: Novel Transformations and Stability Studies

Cristina M. Al Matarneh, Alina Nicolescu, Sergiu Shova, Mircea Apostu, Razvan Puf, Francesca Mocci, Aatto Laaksonen, Ionel I. Mangalagiu, Ramona Danac

Contents:

S1. General synthetic procedures ……………………………………………………..S2

S2. Reprezentative NMR, X-Ray and MS spectra…………………………………….S9

S1. General synthetic procedures

General procedure for synthesis of compound 2

At room temperature and under inert atmosphere, to a dichloromethane (5 mL) suspension containing the cycloimmonium salt (1 mmol) and NEtMI (1.1 mmol), triethylamine (TEA, 3 mmol) was added dropwise over 1 h with vigorous stirring. The reaction mixture was then stirred over the night at room temperature (rt). Methanol (5 mL) was added and the resulting mixture was kept over the night without stirring. The resulted suspension was filtered off to give a solid that was washed with methanol. The crude product was then crystallized from dicloromethane/methanol.

10-Ethyl-12-(4-fluorobenzoyl)-11a,12-dihydro-8aH-pyrrolo[3',4':3,4]pyrrolo[1,2-a][1,10] phenanthroline-9,11(8bH,10H)-dione (2a):

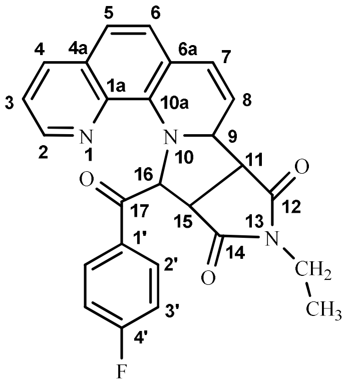

Crystallized from methanol-chloroform1:1, (v/v), yellow crystals, mp = 195-197 °C, yield: 67%. IR (KBr), ν(cm^{-1}): 3058, 3022, 2985, 2955, 2875, 2814, 1776, 1685, 1639, 1588, 1558, 1536 1090.

^{1}H NMR (DMSO-d$_6$, 600.1 MHz, δ(ppm)): 1.04 (3H, t, ^3J = 7 Hz, CH_3-Et), 3.46 (2H, q, ^3J = 7 Hz, CH_2-Et), 3.67-3.71 (2H, m, H-11 and H-15), 5.53-5.55 (1H, m, H-9), 6.07 (1H, dd, ^3J = 10 Hz, ^4J = 2 Hz, H-8), 6.55 (1H, dd, ^3J = 10 Hz, ^4J = 3 Hz, H-7),6.79 (1H, s, H-16), 7.16 (1H, d, ^3J = 8 Hz, H-5), 7.18 (1H, dd, ^3J = 8 Hz, ^4J = 4 Hz, H-3), 7.24 (1H, d, ^3J = 8 Hz, H-6), 7.54 (2H, t, $^3J_{H,H}$ = $^3J_{H,F}$ = 9 Hz, H-3'), 7.73 (1H, dd, ^3J = 4 Hz, ^4J = 2 Hz, H-2), 8.07 (1H, dd, ^3J = 8 Hz, ^4J = 2 Hz, H-4),8.26 (2H, dd, $^3J_{H,H}$ = 8 Hz, $^4J_{H,F}$ = 6 Hz, H-2').

^{13}C NMR (DMSO-d$_6$, 150.9 MHz, δ(ppm)): 12.8 (CH_3-Et), 33.6 (CH_2-Et), 46.3 (CH-9), 6.7 (CH-15), 62.4 (CH-11), 66.1 (CH-16), 116.1 (d, $^2J_{C,F}$ = 23 Hz, CH-3'), 116.7 (CH-5), 120.6 (C-6a), 120.8 (CH-3), 122.0 (CH-8), 126.2 (CH-7), 126.5 (CH-6), 129.1 (C-4a), 131.0 (C-1'), 131.2 (d,

$^3J_{C,F}$ = 9 Hz, CH-2’), 136.3 (CH-4), 137.0 (C-1a), 138.3 (C-10a), 145.5 (CH-2), 164.9 (d, $^1J_{C,F}$ = 251 Hz, C-4’), 176.2 (CO-12), 177.8 (CO-14), 194.1 (CO-17).

General procedure for synthesis of compounds 3, 4, 5 and 6

Adequate aliquots of compound **2a** were immersed in a dichloromethane/methanol solution and kept at room temperature for several days, with progress monitored by TLC. Every two days, each aliquot was subjected to column chromatography, yielding the following results: After 2 days, the major product was compound **3a**; after 4 days, compound **4a** predominated; after 6 days, compound **5a** was identified as the primary product, and after an additional 6 days, compound **6a** appeared. Given the structural resembles in the structures of compounds **5a** and **6a**, we further investigated whether compound **6a** could form from compound **5a**. To test this, compound **5a** was immersed in the same solvent mixture for 2 days and then subjected to column chromatography, confirming that the transformation to compound **6a** indeed occurs.

10-Ethyl-12-(4-fluorobenzoyl)-9H-pyrrolo[3',4':3,4]pyrrolo[1,2-a][1,10]phenanthroline-9,11(10H)-dione (3a):

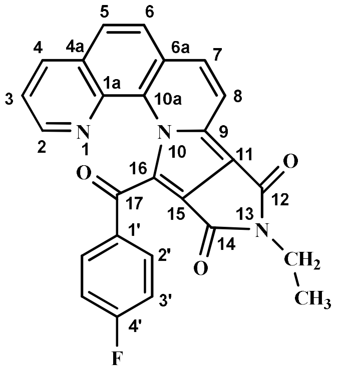

Obtained by general procedure. Purified by column chromatography: CH_2Cl_2→ CH_2Cl_2: MeOH (97:3, v:v), red powder, mp = 232-233 °C, yield: 18%. IR (KBr), ν(cm^{-1}): 3067, 2942, 2350, 1695, 1649, 1622, 1227.

^{1}H NMR ($CDCl_3$, 500.1 MHz, δ(ppm)): 1.21 (3H, t, ^3J = 7 Hz, CH_3-Et), 3.58-3.67 (2H, m, CH_2-Et), 7.30 (2H, t, $^3J_{H,H}$=$^3J_{H,F}$= 9 Hz, H-3’), 7.42 (1H, dd, ^3J = 8 Hz, ^4J = 4 Hz, H-3), 7.77 (1H, d, ^3J = 9 Hz, H-8),7.88 (1H, d, ^3J = 9 Hz, H-5), 7.92 (1H, d, ^3J = 9 Hz, H-6),8.04 (1H, d, ^3J = 9 Hz, H-7), 8.07 (1H, dd, ^3J = 4 Hz, ^4J = 2 Hz, H-2), 8.25 (1H, dd, ^3J = 8 Hz, ^4J = 2 Hz, H-4),8.34 (2H, dd, $^3J_{H,H}$= 9 Hz, $^4J_{H,F}$= 6 Hz, H-2’).

^{13}C NMR ($CDCl_3$, 125.7 MHz, δ(ppm)): 14.1 (CH_3-Et), 33.0 (CH_2-Et), 110.9 (C-11), 115.6 (d, $^2J_{C,F}$ = 23 Hz, CH-3'),118.9 (CH-7), 122.9 (CH-3), 125.8 (CH-5), 125.82 (C-10a), 126.6 (C-15), 126.8 (CH-6), 127.4 (CH-8), 128.1 (C-4a), 128.4 (C-16), 130.2 (C-6a), 131.3 (C-9), 132.9 (d, $^3J_{C,F}$= 9 Hz, CH-2'),133.5 (C-1'), 136.3 (CH-4), 137.2 (C-1a), 146.2 (CH-2), 163.5 (CO-12), 164.1 (CO-14), 165.8 (d, $^1J_{C,F}$ = 255 Hz, C-4'), 182.3 (CO-17).

Methyl 10-(ethylcarbamoyl)-11-(4-fluorobenzoyl)-8a,9,10,11-tetrahydropyrrolo[1,2-a][1,10]phenanthroline-9-carboxylate (4a):

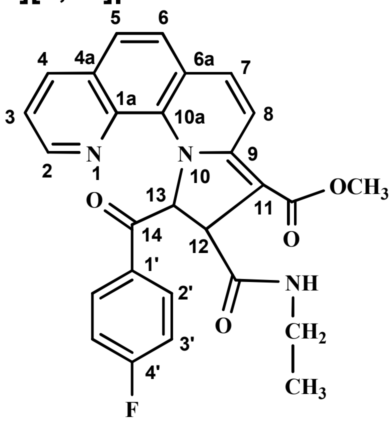

Obtained by general procedure. Purified by column chromatography: CH_2Cl_2→ CH_2Cl_2: MeOH (97:3, v:v), red-violet powder, mp = 211-215 °C, yield: 13%. IR (KBr), ν(cm^{-1}): 3061, 2984, 2938, 2349, 1751, 1709, 1645, 1537, 1450.

^1H NMR ($CDCl_3$, 400.1 MHz, δ(ppm)): 1.14 (3H, t, ^3J = 7 Hz, CH_3-Et), 3.25-3.37 (2H, m, CH_2-Et), 3.25-3.37 (2H, m, CH_2-Et), 3.80 (3H, s, OCH_3), 3.98 (1H, d, ^3J = 4 Hz, H-12), 7.23 (1H, dd, ^3J = 8 Hz, ^4J = 4 Hz, H-3), 7.24 (2H, t, $^3J_{H,H}$= $^3J_{H,F}$ = 9 Hz, H-3'),7.38 (1H, d, ^3J = 9 Hz, H-5), 7.46 (1H, d, ^3J = 9 Hz, H-6), 7.50 (1H, d, ^3J = 9 Hz, H-8), 7.74-7.76 (2H, m, H-7 and NH), 8.00 (1H, dd, ^3J = 8 Hz, ^4J = 2 Hz, H-4), 8.07 (1H, dd, ^3J = 4 Hz, ^4J = 2 Hz, H-2), 8.28 (2H, dd, $^3J_{H,H}$= 9 Hz, $^4J_{H,F}$ = 6 Hz, H-2'), 8.32 (1H, d, ^3J = 4 Hz, H-13).

^{13}C NMR ($CDCl_3$, 100.6 MHz, δ(ppm)): 14.7 (CH_3-Et), 34.8 (CH_2-Et), 49.3 (CH-12), 50.8 (OCH_3), 69.7 (CH-13), 87.6 (C-11), 116.0 (d, $^2J_{C,F}$ = 23 Hz, CH-3'),119.6 (CH-7), 121.4 (CH-5), 121.9 (CH-3 and C-6a), 126.5 (CH-6), 130.2 (C-1'), 130.4 (C-4a), 132.0 (d, $^3J_{C,F}$= 9 Hz, CH-2'), 135.8 (C-10a), 136.3 (CH-4), 136.8 (CH-8), 137.9 (C-1a), 146.5 (CH-2), 154.5 (C-9), 165.8 (d, $^1J_{C,F}$ = 255 Hz, C-4'), 167.9 (COO-11), 171.1 (CONH-12), 190.6 (CO-14).

(Z)-1-ethyl-3-(2-(4-fluorophenyl)-2-oxoethyl)-4-(1,10-phenanthrolin-2(1H)-ylidene) pyrrolidine-2,5-dione (5a):

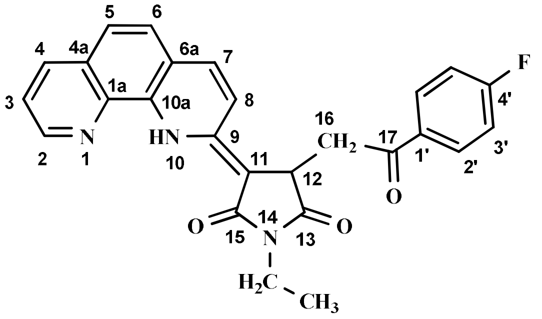

Obtained by general procedure. Purified by column chromatography: CH_2Cl_2→ CH_2Cl_2: MeOH (97:3, v:v), red crystals, mp = 220-222 °C, yield: 13%. IR (KBr), ν(cm^{-1}): 3061, 2984, 2938, 2349, 1751, 1709, 1645, 1537, 1450.

^{1}H NMR (DMSO-d$_6$, 400.1 MHz, δ(ppm)): 1.17 (3H, t, ^3J = 7 Hz, CH_3-Et), 3.48-3.57 (3H, m, CH_2-Et and H-16B), 3.88 (1H, dd, ^3J = 5 Hz, ^3J = 4 Hz, H-12), 3.98 (1H, dd, ^2J = 18 Hz, ^4J = 5 Hz, H-16A), 7.06 (1H, dd, ^3J = 9 Hz, ^4J = 2 Hz, H-8), 7.32 (2H, t, $^3J_{H,H}$= $^3J_{H,F}$ = 9 Hz, H-3'),7.63 (1H, d, ^3J = 9 Hz, H-5), 7.70 (1H, d, ^3J = 9 Hz, H-6), 7.74 (1H, dd, ^3J = 8 Hz, ^4J = 4 Hz, H-3), 7.81 (1H, d, ^3J = 9 Hz, H-7), 8.04 (2H, dd, $^3J_{H,H}$= 9 Hz, $^4J_{H,F}$ = 6 Hz, H-2'), 8.45 (1H, dd, ^3J = 8 Hz, ^4J = 2 Hz, H-4), 9.04 (1H, dd, ^3J = 4 Hz, ^4J = 2 Hz, H-2), 13.5 (1H, s, NH).

^{13}C NMR (DMSO-d$_6$, 150.9 MHz, δ(ppm)): 13.2 (CH_3-Et), 32.2 (CH_2-Et), 38.1 (CH_2-16), 38.5 (CH-12), 49.3 (CH-12), 87.8 (C-11), 115.7 (d, $^2J_{C,F}$ = 23 Hz, CH-3'), 118.7 (C-6a), 120.0 (CH-8), 120.7 (CH-5), 123.7 (CH-3), 125.5 (CH-6), 128.7 (C-4a), 131.0 (d, $^3J_{C,F}$= 9 Hz, CH-2'), 133.1 (C-1'), 134.7 (C-10a), 135.9 (CH-7), 136.0 (C-1a), 136.6 (CH-4), 143.8 (C-9), 149.9 (CH-2), 165.6 (d, $^1J_{C,F}$ = 270 Hz, C-4'), 171.0 (CO-13), 177.0 (CO-15), 196.2 (CO-17).

(3Z,4Z)-1-ethyl-3-(2-(4-fluorophenyl)-2-oxoethylidene)-4-(1,10-phenanthrolin-2(1H)-ylidene)pyrrolidine-2,5-dione (6a):

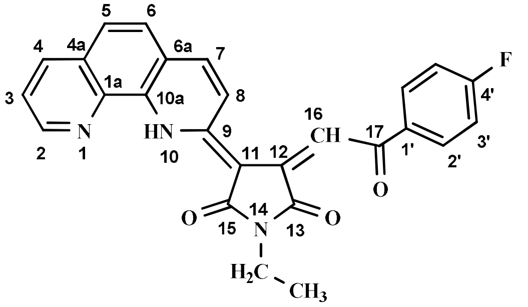

Obtained by general procedure. Purified by column chromatography: CH_2Cl_2→ CH_2Cl_2: MeOH (97:3, v:v), red crystals, mp = 215-216 °C, yield: 13%. IR (KBr), ν(cm^{-1}): 3064, 2920, 2850,

2359, 2162, 2015, 1973, 1741, 1732, 1666, 1625, 1595, 1537, 1504, 1489, 1442, 1402, 1373, 1344, 1229, 1215, 1195, 1147, 1103, 1072, 1029, 1010, 962, 879, 835, 813, 761, 719, 601, 578, 422.

^{1}H NMR (DMSO-d$_6$, 400.1 MHz, δ(ppm)): 1.21 (3H, t, ^3J = 7 Hz, CH_3-Et), 3.66 (2H, q, ^3J = 7 Hz, CH_2-Et), 6.88 (1H, bs, H-8), 7.28 (1H, s, H-16), 7.39 (2H, t, $^3J_{H,H}$= $^3J_{H,F}$ = 9 Hz, H-3'),7.88 (1H, dd, ^3J = 8 Hz, ^4J = 4 Hz, H-3), 7.99 (2H, bs, H-5 and H-6), 8.11 (2H, dd, $^3J_{H,H}$= 9 Hz, $^4J_{H,F}$ = 6 Hz, H-2'), 8.45 (1H, d, ^3J = 9 Hz, H-7), 8.61 (1H, dd, ^3J = 8 Hz, ^4J = 2 Hz, H-4), 9.06 (1H, d, ^3J = 4 Hz, H-2), 14.2 (1H, s, NH).

^{13}C NMR (DMSO-d$_6$, 100.6 MHz, δ(ppm)): 13.6 (CH_3-Et), 32.4 (CH_2-Et), 91.2 (C-11), 106.4 (CH-16), 115.7 (d, $^2J_{C,F}$ = 23 Hz, CH-3'), 121.4 (C-6a), 124.4 (CH-5), 124.6 (CH-3), 125.1 (CH-6), 125.2 (CH-8), 128.7 (C-4a), 130.5 (d, $^3J_{C,F}$= 9 Hz, CH-2'), 133.3 (C-10a), 136.2 (C-1'),136.8 (CH-4), 137.0 (C-12), 137.4 (C-1a), 137.7 (CH-7), 145.9 (C-9), 150.4 (CH-2), 164.3 (d, $^1J_{C,F}$ = 250 Hz, C-4'), 169.9 (CO-13),172.0 (CO-15), 186.0 (CO-17).

^{1}H NMR ($CDCl_3$, 400.1 MHz, δ(ppm)): 1.31 (3H, t, ^3J = 7 Hz, CH_3-Et), 3.80 (2H, q, ^3J = 7 Hz, CH_2-Et), 6.57 (1H, bs, H-8), 7.18 (2H, t, $^3J_{H,H}$= $^3J_{H,F}$ = 9 Hz, H-3'), 7.43 (1H, bs, H-16), 7.67 (1H, bs, H-3), 7.73 (2H, s, H-5 and H-6), 8.09-8.15 (3H, m, H-7 and H-2'), 8.29 (1H, dd, ^3J = 8 Hz, ^4J = 2 Hz, H-4), 9.13 (1H, bs, H-2), 14.94 (1H, s, NH).

12-(4-Chlorobenzoyl)-10-ethyl-11a,12-dihydro-8aH-pyrrolo[3',4':3,4]pyrrolo[1,2-a][1,10]phenanthroline-9,11(8bH,10H)-dione (2b):

^{1}H NMR ($CDCl_3$, 500.1 MHz, δ(ppm)): 1.15 (3H, t, ^3J = 7 Hz, CH_3-Et), 3.42 (1H, dd, ^3J = 8 Hz, ^4J = 1 Hz, H-11), 3.57 (2H, q, ^3J = 7 Hz, CH_2-Et), 3.58 (1H, dd, ^3J = 7 Hz, ^4J = 2 Hz, H-15), 5.81 (1H, dt, ^3J = 8 Hz, ^4J = 2.0 Hz, H-9), 6.13 (1H, dd, ^3J = 10 Hz, ^4J = 2 Hz, H-8), 6.47 (1H, dd, ^3J = 10 Hz, ^4J = 2 Hz, H-7), 6.89 (1H, d, ^3J = 1 Hz, H-16), 6.99 (1H, dd, ^3J = 8 Hz, ^4J = 4 Hz, H-3), 7.03 (1H, d, ^3J = 8 Hz, H-5), 7.12 (1H, d, ^3J = 8 Hz, H-6), 7.58 (2H, d, ^3J = 9 Hz, H-3'), 7.70 (1H, dd, ^3J = 4 Hz, ^4J = 2 Hz, H-2), 7.84 (1H, dd, ^3J = 8 Hz, ^4J = 2 Hz, H-4), 8.25 (2H, d, ^3J = 9 Hz, H-2').

12-(4-Bromobenzoyl)-10-ethyl-11a,12-dihydro-8aH-pyrrolo[3',4':3,4]pyrrolo[1,2-a][1,10]phenanthroline-9,11(8bH,10H)-dione (2c):

^{1}H NMR ($CDCl_3$, 400.1 MHz, δ (ppm)): 1.16 (3H, t, ^3J = 8 Hz, CH_3-Et), 3.42 (1H, dd, ^3J = 8 Hz, ^4J = 1.5 Hz, H-11), 3.57 (2H, q, ^3J = 8 Hz, CH_2-Et), 3.61 (1H, dd, ^3J = 7 Hz, ^4J = 1 Hz, H-15),

5.83 (1H, dt, ^3J = 8 Hz, ^4J = 2.0 Hz, H-9), 6.15 (1H, dd, ^3J = 10 Hz, ^4J = 2 Hz, H-8), 6.48 (1H, dd, ^3J = 10 Hz, ^4J = 3 Hz, H-7), 6.89 (1H, d, ^3J = 1 Hz, H-16), 7.01 (1H, dd, ^3J = 8 Hz, ^4J = 4 Hz, H-3), 7.05 (1H, d, ^3J = 8 Hz, H-5), 7.13 (1H, d, ^3J = 8 Hz, H-6), 7.71 (1H, dd, ^3J = 4 Hz, ^4J = 2 Hz, H-2), 7.75 (2H, d, ^3J = 9 Hz, H-3'), 7.86 (1H, dd, ^3J = 8 Hz, ^4J = 2 Hz, H-4), 8.18 (2H, d, ^3J = 9 Hz, H-2').

10-Ethyl-12-(4-methoxybenzoyl)-11a,12-dihydro-8aH-pyrrolo[3',4':3,4]pyrrolo[1,2-a][1,10]phenanthroline-9,11(8bH,10H)-dione (2d):

^{1}H NMR ($CDCl_3$, 400.1 MHz, δ (ppm)): 1.16 (3H, t, ^3J = 8 Hz, CH_3-Et), 3.45 (1H, dd, ^3J = 8 Hz, ^4J = 1 Hz, H-11), 3.54 (1H, t, ^3J = 8 Hz, H-15), 3.58 (2H, q, ^3J = 8 Hz, CH_2-Et), 3.94 (3H, s, OCH_3), 5.84 (1H, dt, ^3J = 8 Hz, ^4J = 2.0 Hz, H-9), 6.12 (1H, dd, ^3J = 10 Hz, ^4J = 2 Hz, H-8), 6.47 (1H, dd, ^3J = 10 Hz, ^4J = 3 Hz, H-7), 6.98 (1H, d, ^3J = 1 Hz, H-16), 7.00 (1H, dd, ^3J = 8 Hz, ^4J = 4 Hz, H-3), 7.03 (1H, d, ^3J = 8 Hz, H-5), 7.08 (2H, d, ^3J = 9 Hz, H-3'), 7.11 (1H, d, ^3J = 8 Hz, H-6), 7.79 (1H, dd, ^3J = 4 Hz, ^4J = 2 Hz, H-2), 7.84 (1H, dd, ^3J = 8 Hz, ^4J = 2 Hz, H-4), 8.29 (2H, d, ^3J = 9 Hz, H-2').

10-Ethyl-12-(4-nitrobenzoyl)-11a,12-dihydro-8aH-pyrrolo[3',4':3,4]pyrrolo[1,2-a][1,10]phenanthroline-9,11(8bH,10H)-dione (2e):

^{1}H NMR ($CDCl_3$, 400.1 MHz, δ (ppm)): 1.18 (3H, t, ^3J = 8 Hz, CH_3-Et), 3.44 (1H, dd, ^3J = 8 Hz, ^4J = 1.5 Hz, H-11), 3.58-3.64 (3H, m, H-15 and CH_2-Et), 5.84 (1H, dt, ^3J = 8 Hz, ^4J = 2.0 Hz, H-9), 6.18 (1H, dd, ^3J = 10 Hz, ^4J = 2 Hz, H-8), 6.50 (1H, dd, ^3J = 10 Hz, ^4J = 3 Hz, H-7), 6.86 (1H, d, ^3J = 1 Hz, H-16), 7.00 (1H, dd, ^3J = 8 Hz, ^4J = 4 Hz, H-3), 7.08 (1H, d, ^3J = 8 Hz, H-5), 7.16 (1H, d, ^3J = 8 Hz, H-6), 7.56 (1H, dd, ^3J = 4 Hz, ^4J = 2 Hz, H-2), 7.88 (1H, dd, ^3J = 8 Hz, ^4J = 2 Hz, H-4), 8.46 (2H, d, ^3J = 9 Hz, H-3'), 8.49 (2H, d, ^3J = 9 Hz, H-2').

(3Z,4Z)-1-ethyl-3-(2-(4-chlorophenyl)-2-oxoethylidene)-4-(1,10-phenanthrolin-2(1H)-ylidene)pyrrolidine-2,5-dione (6b):

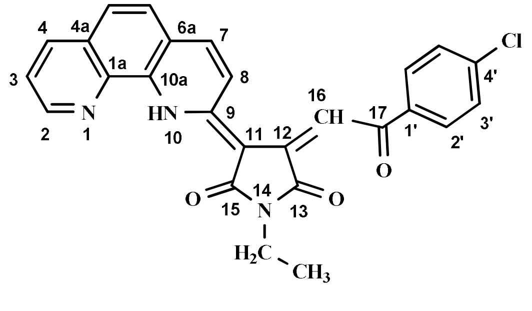

^1H NMR ($CDCl_3$, 400.1 MHz, δ(ppm)): 1.31 (3H, t, ^3J = 7 Hz, CH_3-Et), 3.80 (2H, q, ^3J = 7 Hz, CH_2-Et), 6.61 (1H, bs, H-8), 7.42 (1H, bs, H-16), 7.48 (2H, d, ^3J = 8 Hz, H-3'), 7.64-7.69 (1H, m, H-3), 7.74 (2H, s, H-5 and H-6), 8.05 (2H, d, ^3J = 8 Hz, H-2'), 8.12 (1H, d, ^3J = 8 Hz, H-7), 8.29 (1H, d, ^3J = 8 Hz, H-4), 9.11 (1H, bs, H-2), 14.95 (1H, s, NH).

(3Z,4Z)-1-ethyl-3-(2-(4-bromophenyl)-2-oxoethylidene)-4-(1,10-phenanthrolin-2(1H)-ylidene)pyrrolidine-2,5-dione (6c):

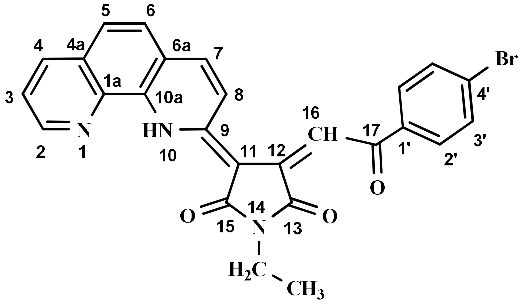

^1H NMR ($CDCl_3$, 400.1 MHz, δ(ppm)): 1.31 (3H, t, ^3J = 7 Hz, CH_3-Et), 3.80 (2H, q, ^3J = 7 Hz, CH_2-Et), 6.60 (1H, bs, H-8), 7.41 (1H, bs, H-16), 7.64-7.70 (3H, m, H-3 and H-3'), 7.74 (2H, s, H-5 and H-6), 7.97 (2H, d, ^3J = 8 Hz, H-2'), 8.12 (1H, d, ^3J = 8 Hz, H-7), 8.29 (1H, d, ^3J = 8 Hz, H-4), 9.10 (1H, bs, H-2), 14.92 (1H, s, NH).

(3Z,4Z)-1-ethyl-3-(2-(4-methoxyphenyl)-2-oxoethylidene)-4-(1,10-phenanthrolin-2(1H)-ylidene)pyrrolidine-2,5-dione (6d):

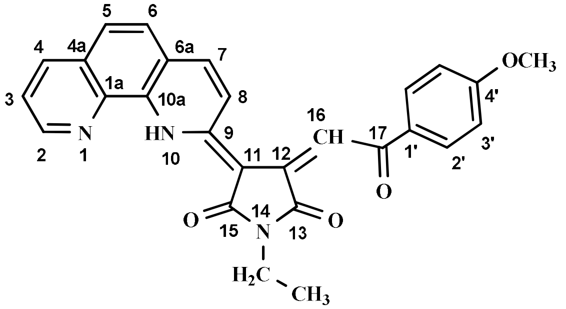

^1H NMR ($CDCl_3$, 400.1 MHz, δ(ppm)): 1.31 (3H, t, ^3J = 7 Hz, CH_3-Et), 3.80 (2H, q, ^3J = 7 Hz, CH_2-Et), 3.91 (3H, s, OCH_3), 6.58 (1H, bs, H-8), 7.01 (2H, d, ^3J = 8 Hz, H-3'), 7.48 (1H, bs, H-16), 7.66 (1H, bs, H-3), 7.70 (2H, s, H-5 and H-6), 8.04 (1H, d, ^3J = 8 Hz, H-7), 8.12 (2H, d, ^3J = 8 Hz, H-2'), 8.27 (1H, d, ^3J = 8 Hz, H-4), 9.14 (1H, bs, H-2), 14.90 (1H, s, NH).

S2. Reprezentative NMR, X-Ray and MS spectra

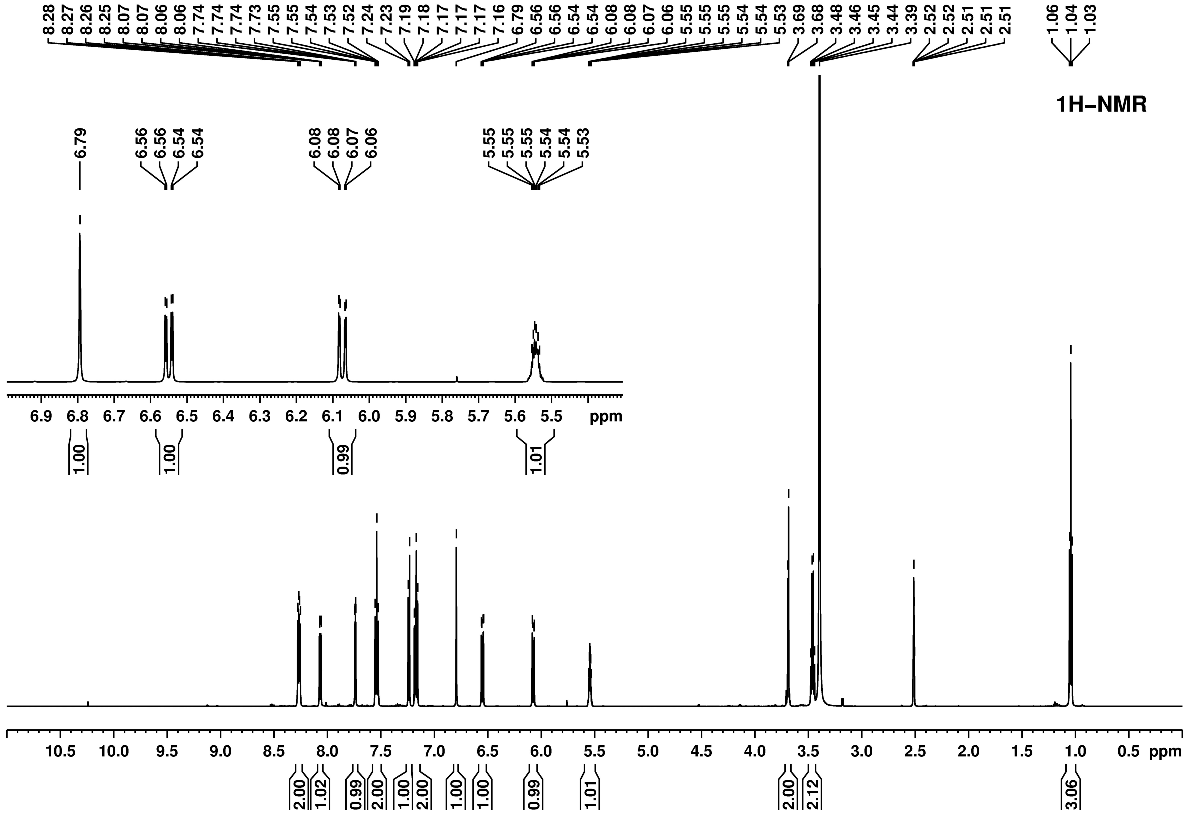

Figure S1. The ^{1}H-NMR spectrum corresponding to compound **2a**, recorded in DMSO-d$_6$ at 600 MHz.

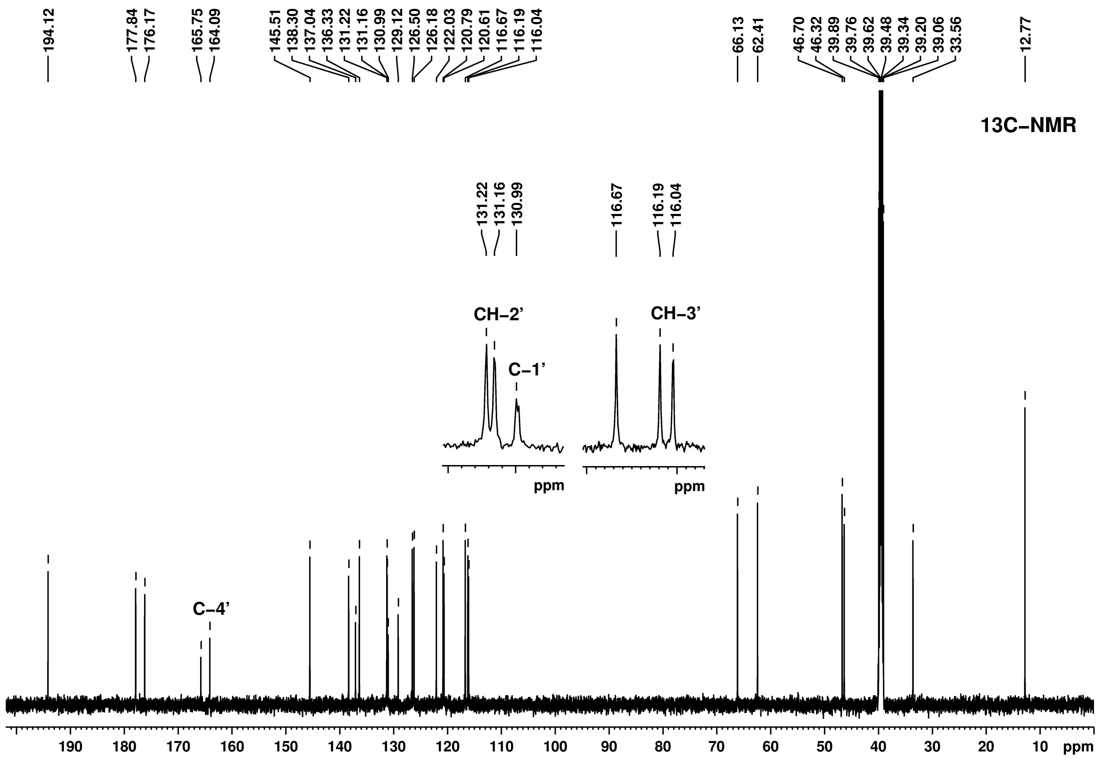

Figure S2. The ^{13}C-NMR spectrum corresponding to compound **2a**, recorded in DMSO-d$_6$ at 150 MHz. The doublets due to carbon-fluorine couplings are annotated on the figure.

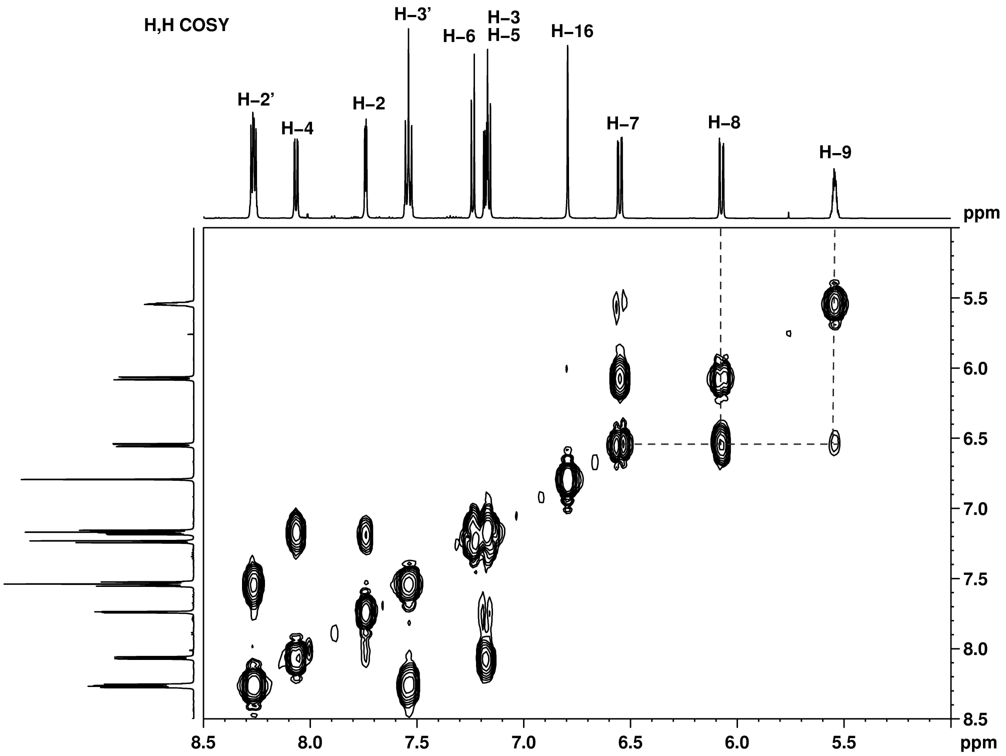

Figure S3. The low field region of the H,H-COSY spectrum corresponding to compound **2a**, recorded in DMSO-d_6 at 600 MHz. As an example, the H-7, H-8 and H-9 spin system is annotated on the figure.

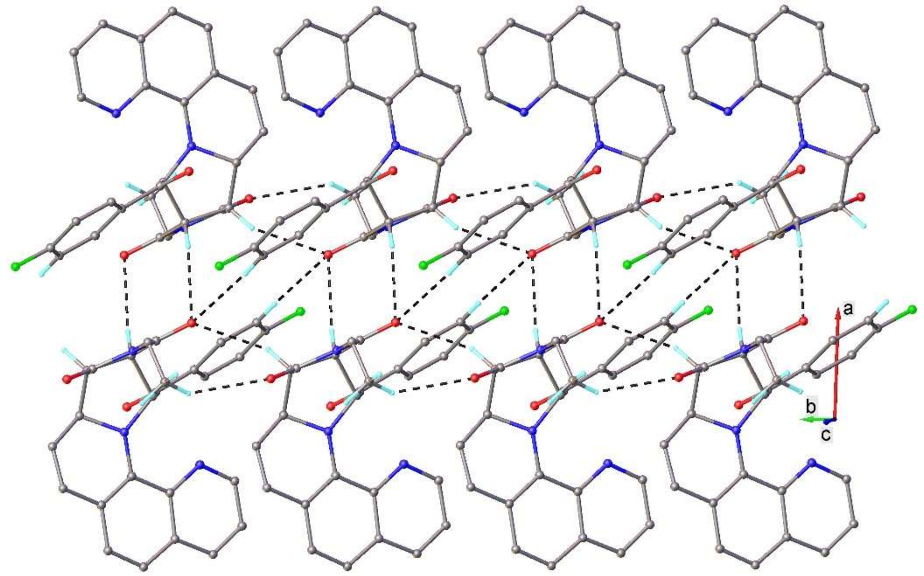

Figure S4. 1D supramolecular array in the crystal structure of **2a** showing the role of intermolecular hydrogen bonding. H-atoms non-involved in H-bonds are omitted for clarity. Hydrogen bond parameters: C20-H···O3 [C20-H 0.98 Å, H···O3 2.53 Å, C20···O3(–*x*, 1 –*y*, –*z*) 3.467(4) Å, ∠C20HO3 159.7°; C21-H···O3 [C21-H 0.98 Å, H···O3 2.50 Å, C21···O3(*x*, *y*– 1, *z*) 3.369(5) Å, ∠C21HO3 147.9°; C25-H···O2 [C20-H 0.96 Å, H···O2 2.59 Å, C25···O2(*x*, 1 + *y*, *z*) 3.346(7) Å, ∠C25HO2 136.3°; C26-H···O3 [C26-H 0.93 Å, H···O3 2.51 Å, C26···O3(*x*, –*y*, –*z*) 3.440(5) Å, ∠C25HO2 173.4°

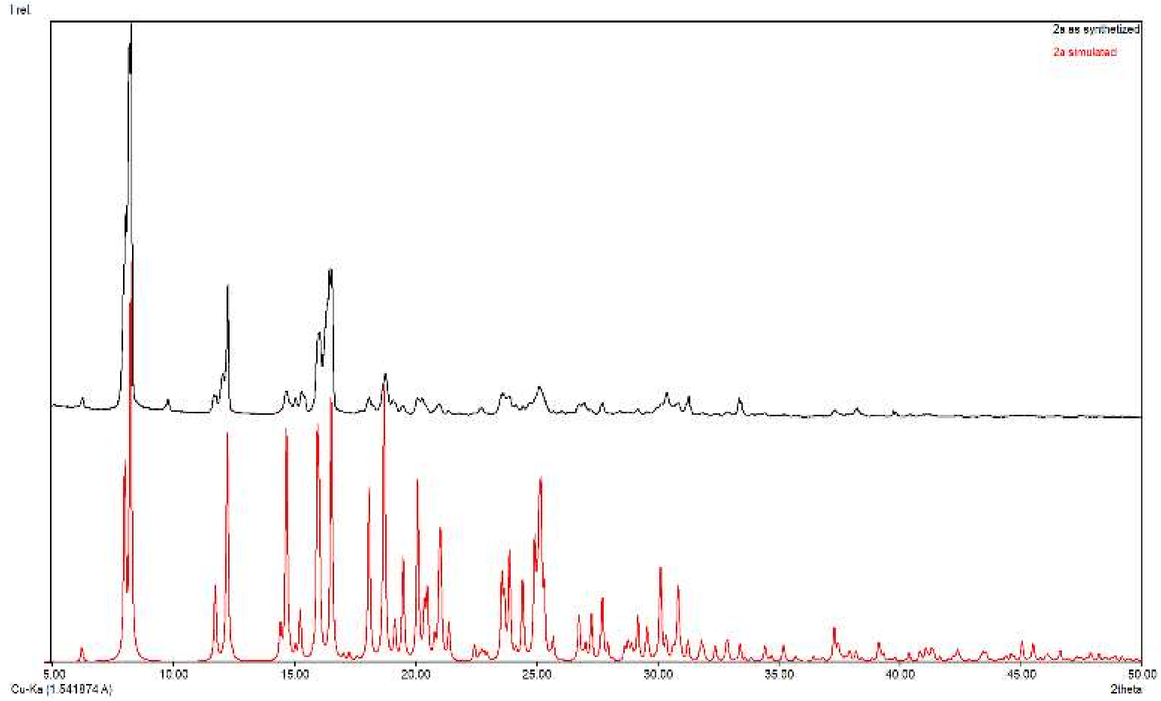

Figure S5. The PXRD patterns of the derivative **2a** showing the similitude between simulated and experimental diffraction peaks.

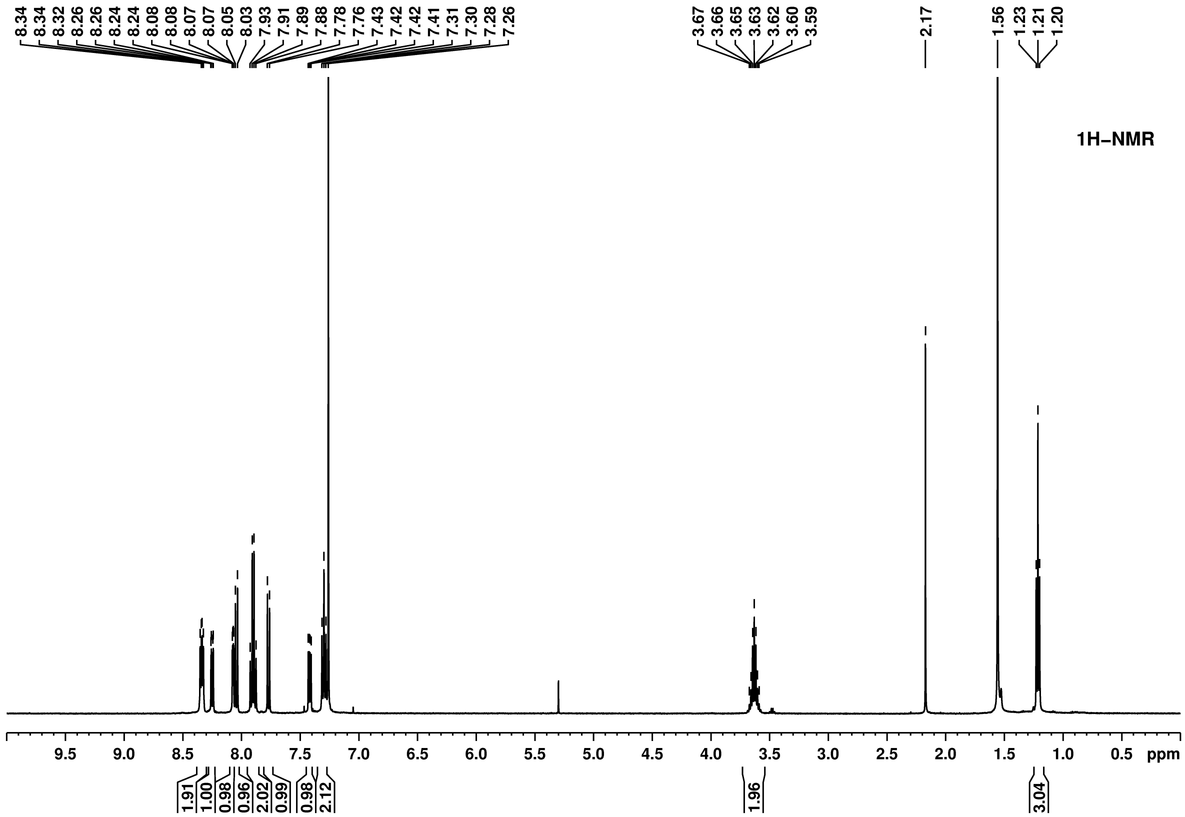

Figure S6. The ^{1}H-NMR spectrum corresponding to compound **3a**, recorded in $CDCl_3$ at 500 MHz.

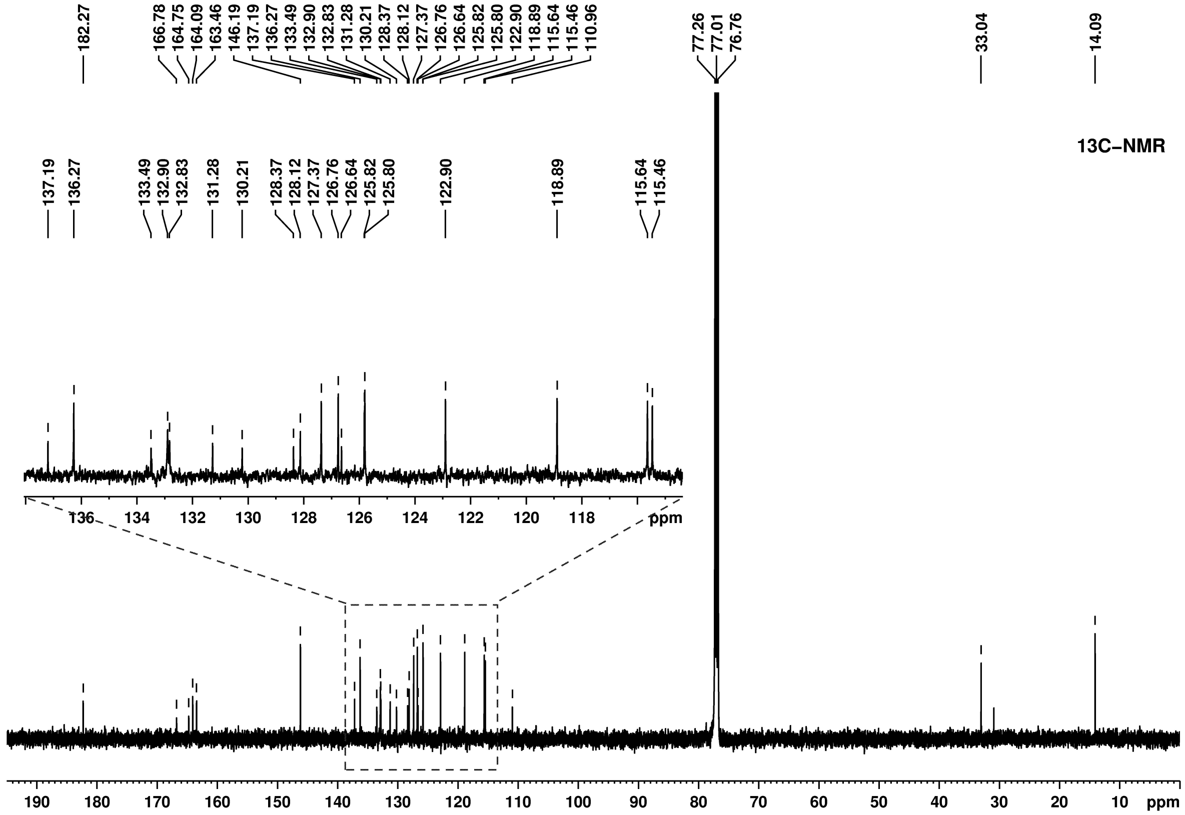

Figure S7. The ^{13}C-NMR spectrum corresponding to compound **3a**, recorded in $CDCl_3$ at 125 MHz.

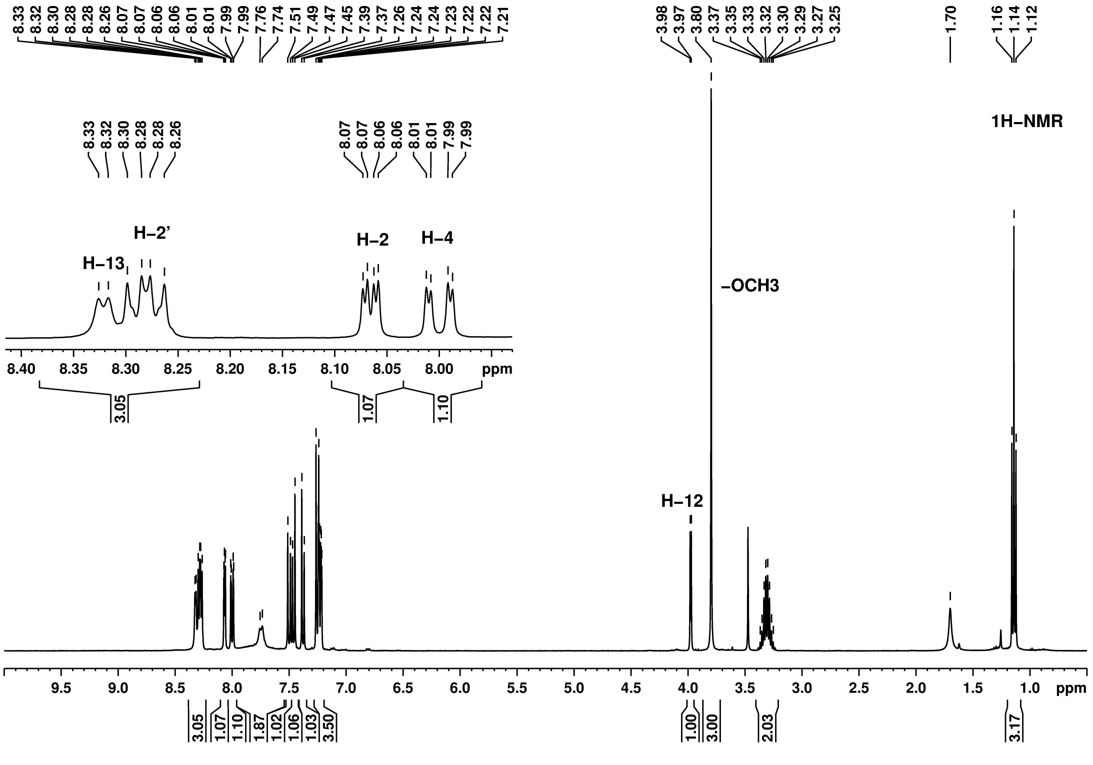

Figure S8. The ^{1}H-NMR spectrum corresponding to compound **4a**, recorded in $CDCl_3$ at 400 MHz. The signals that support the structural particularities are annotated on the figure.

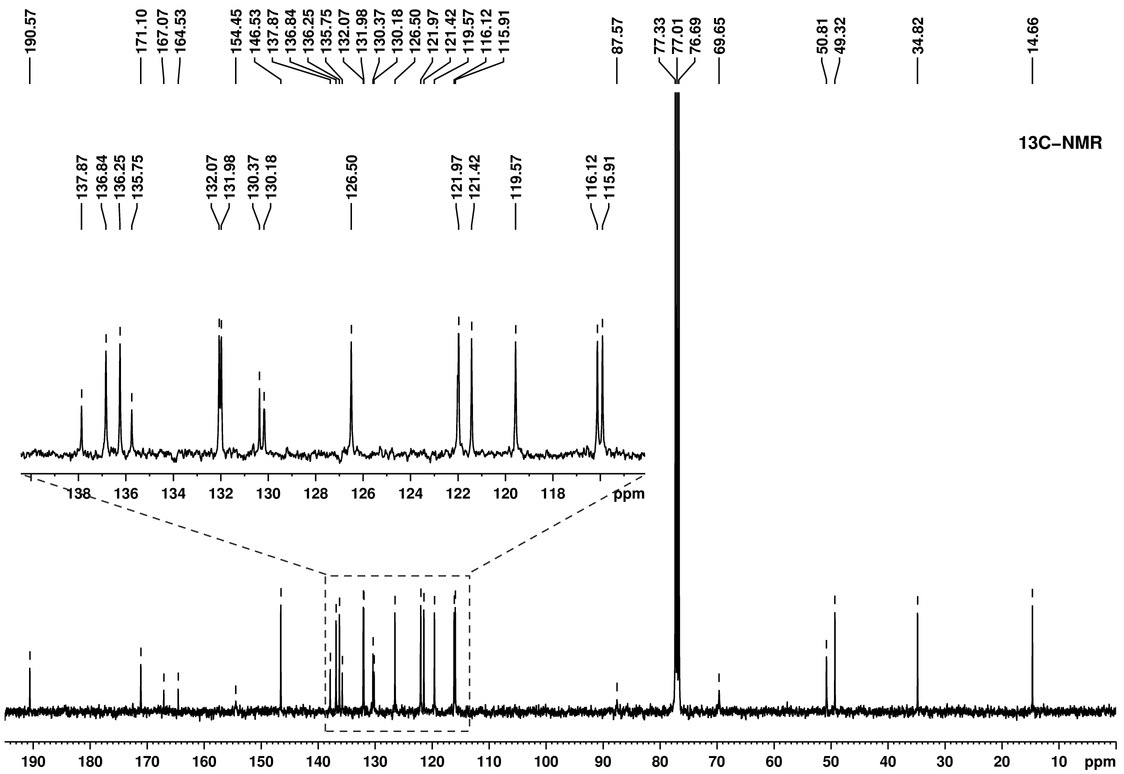

Figure S9. The ^{13}C-NMR spectrum corresponding to compound **4a**, recorded in $CDCl_3$ at 100 MHz

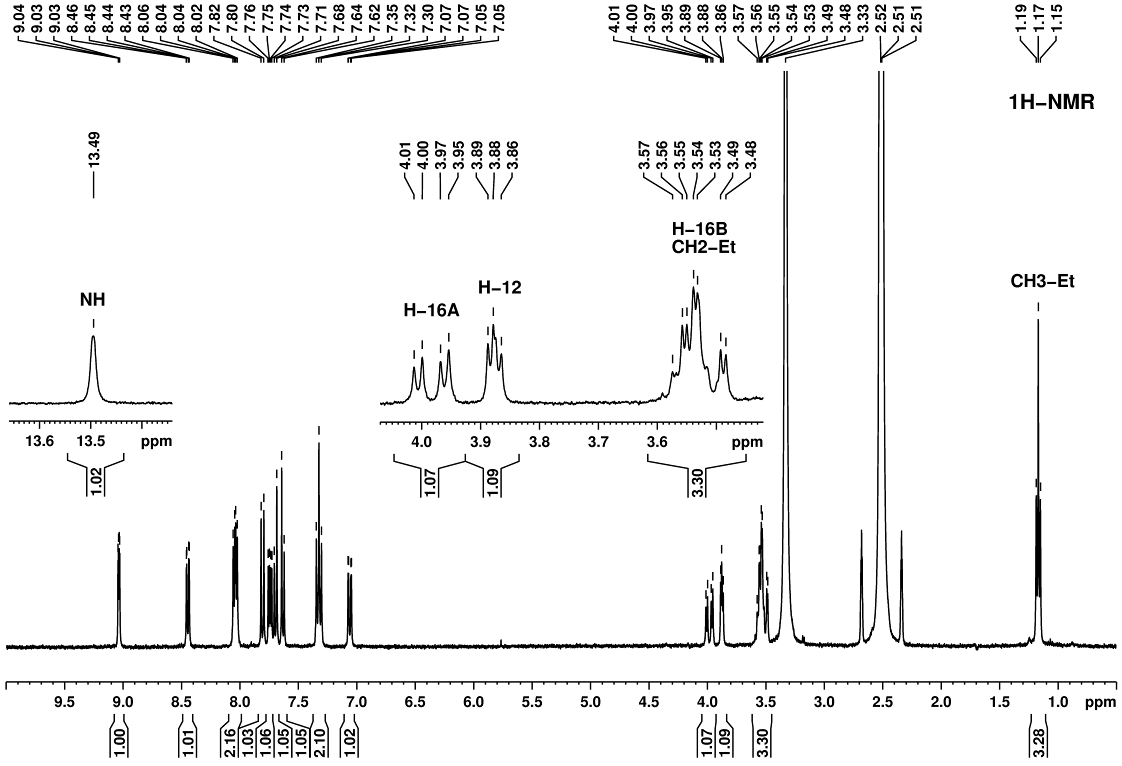

Figure S10. The ^{1}H-NMR spectrum corresponding to compound **5a**, recorded in DMSO-d_6 at 400 MHz. The signals that support the structural particularities are annotated on the figure.

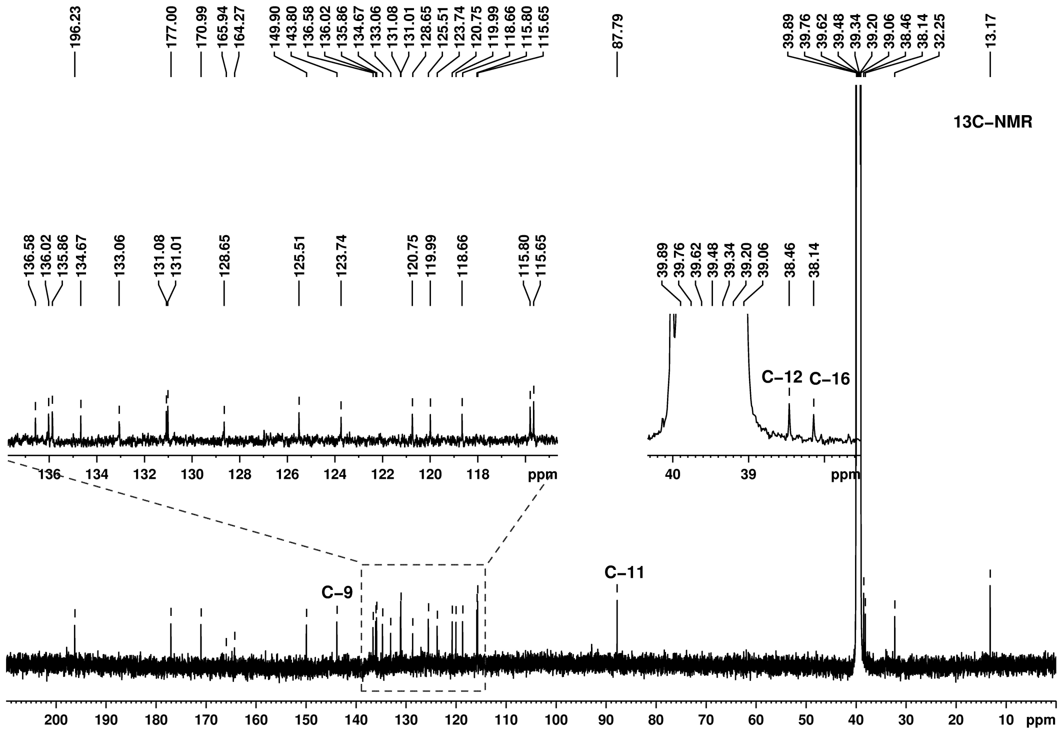

Figure S11. The ^{13}C-NMR spectrum corresponding to compound **5a**, recorded in DMSO-d_6 at 150 MHz. The signals that support the structural particularities are annotated on the figure.

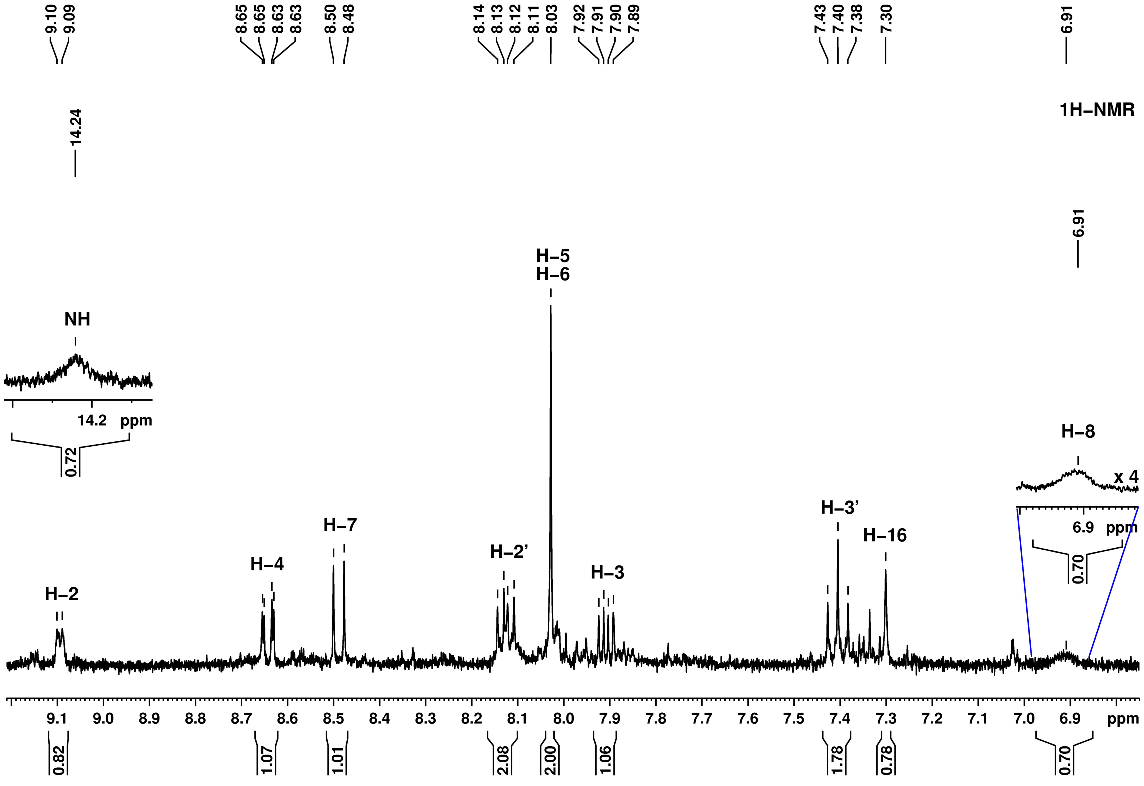

a)

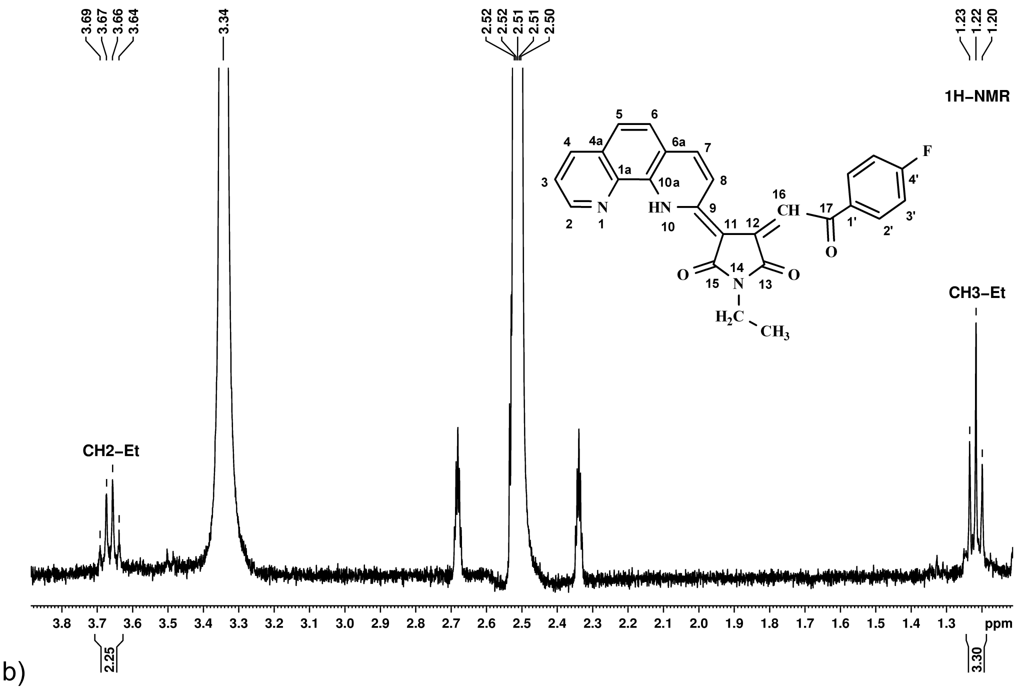

b)

Figure S12. a) Low field region (6.8-9.2 ppm) and **b)** high field region (1.0-3.8 ppm) of the ^{1}H-NMR spectrum corresponding to compound **6a**, recorded in DMSO-d_6 at 400 MHz. The signals that support the structural particularities are annotated on the figure. For a better visualization, NH's signal is included in the insert.

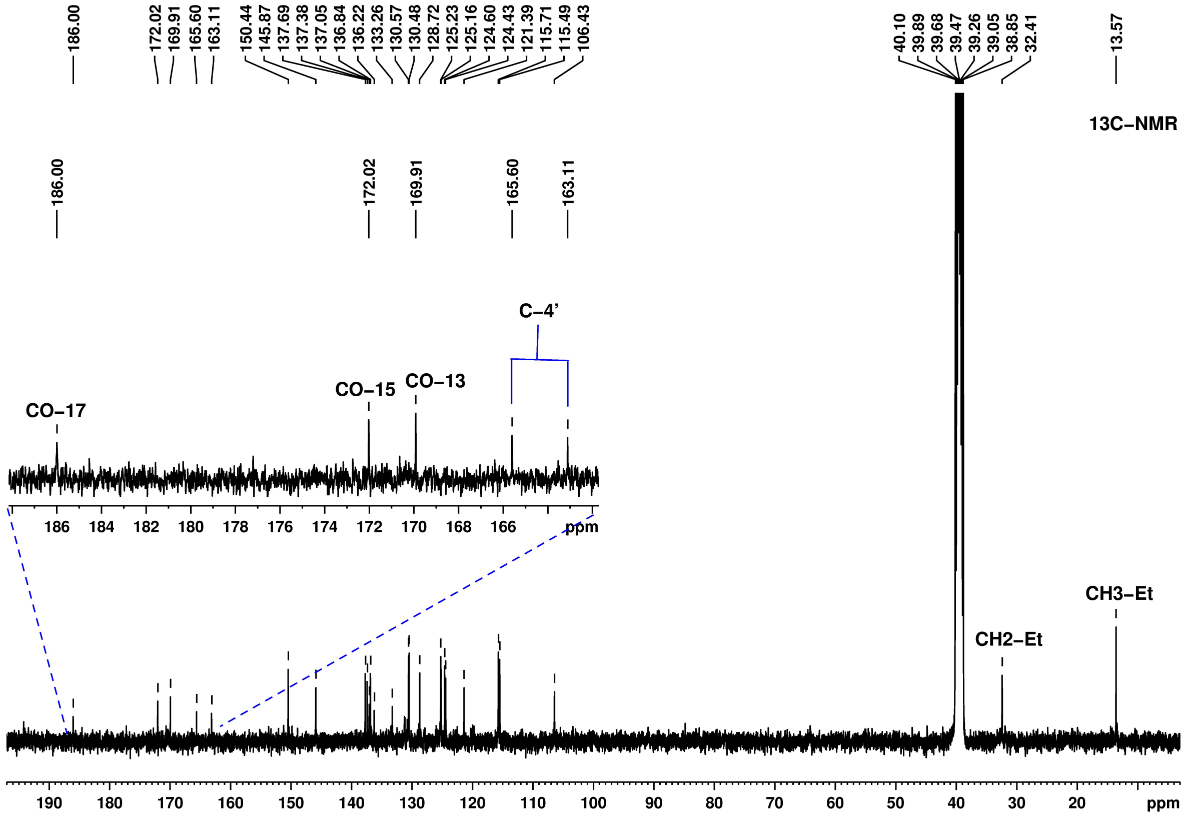

a)

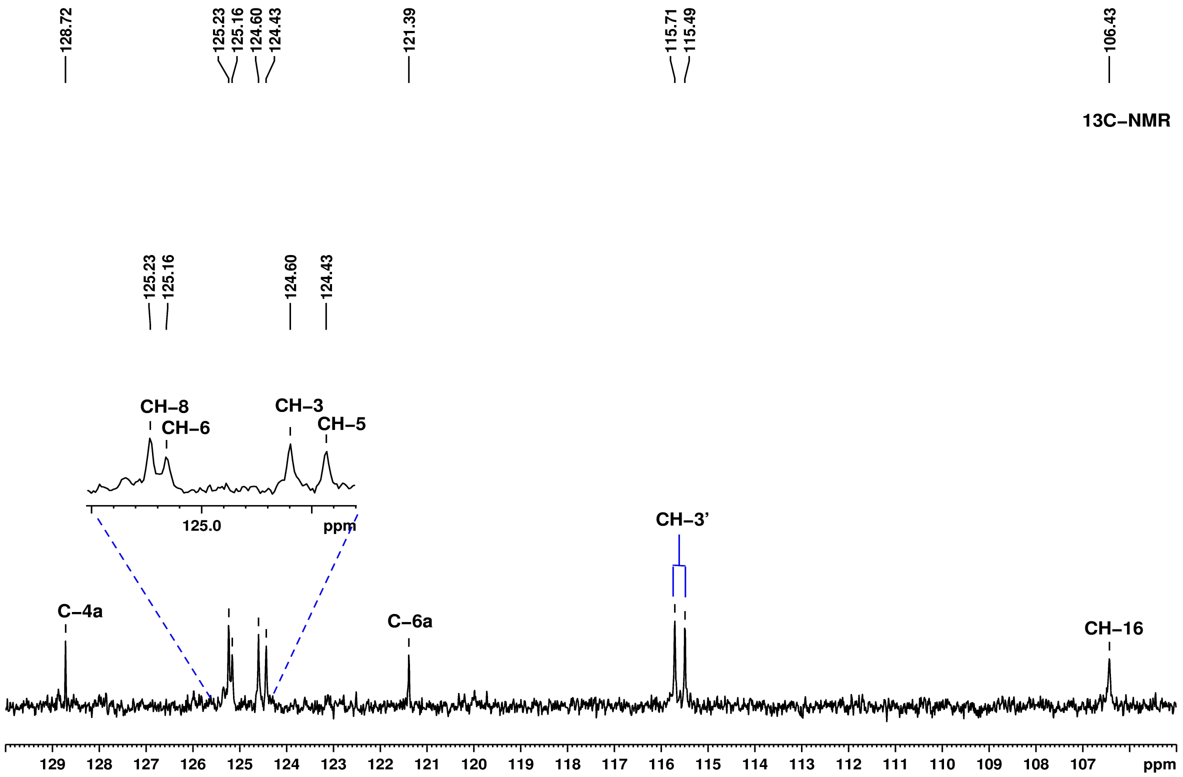

b)

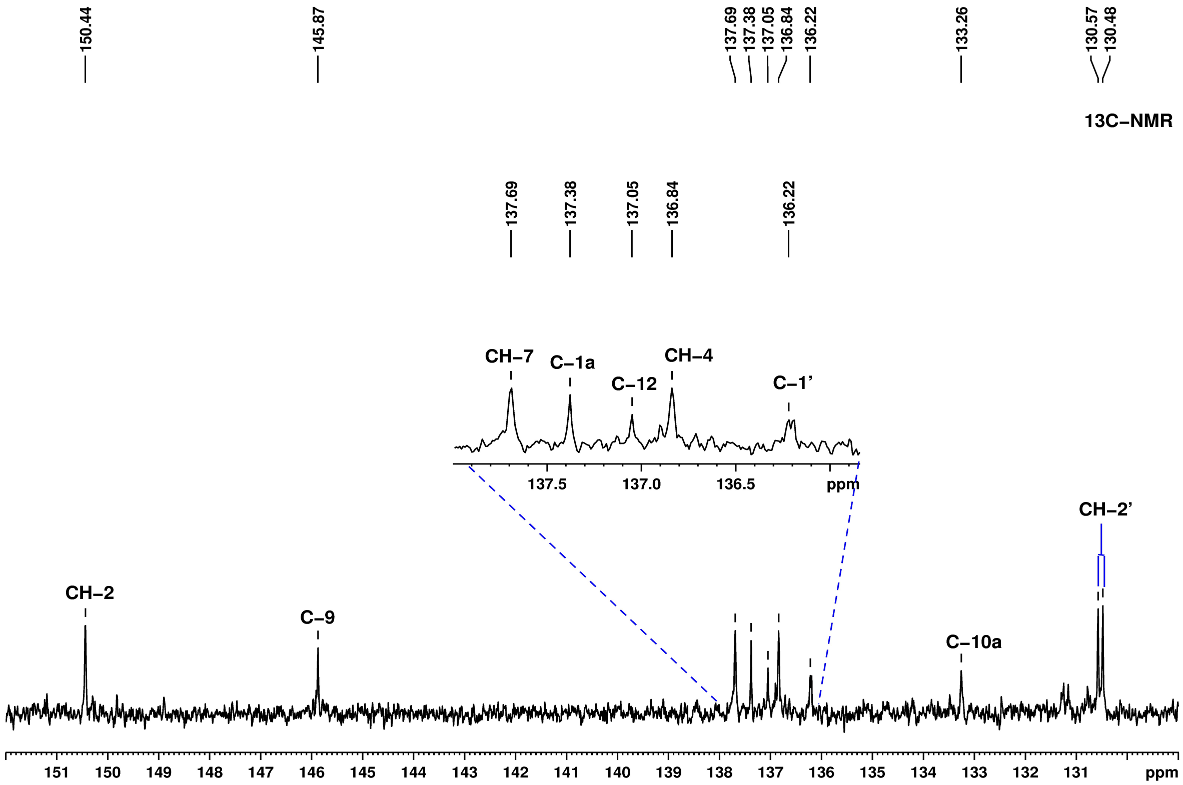

c)

Figure S13. **a)** Full ^{13}C-NMR spectrum corresponding to compound **6a**, recorded in DMSO-d$_6$ at 100 MHz. The signals that support the structural particularities are annotated on the figure. For a better visualization of the number of signals and of the doublets generated by the ^{13}C-^{19}F couplings, the low field region was divided in two details 105-130 ppm (**b**) and 130-152 ppm (**c**).

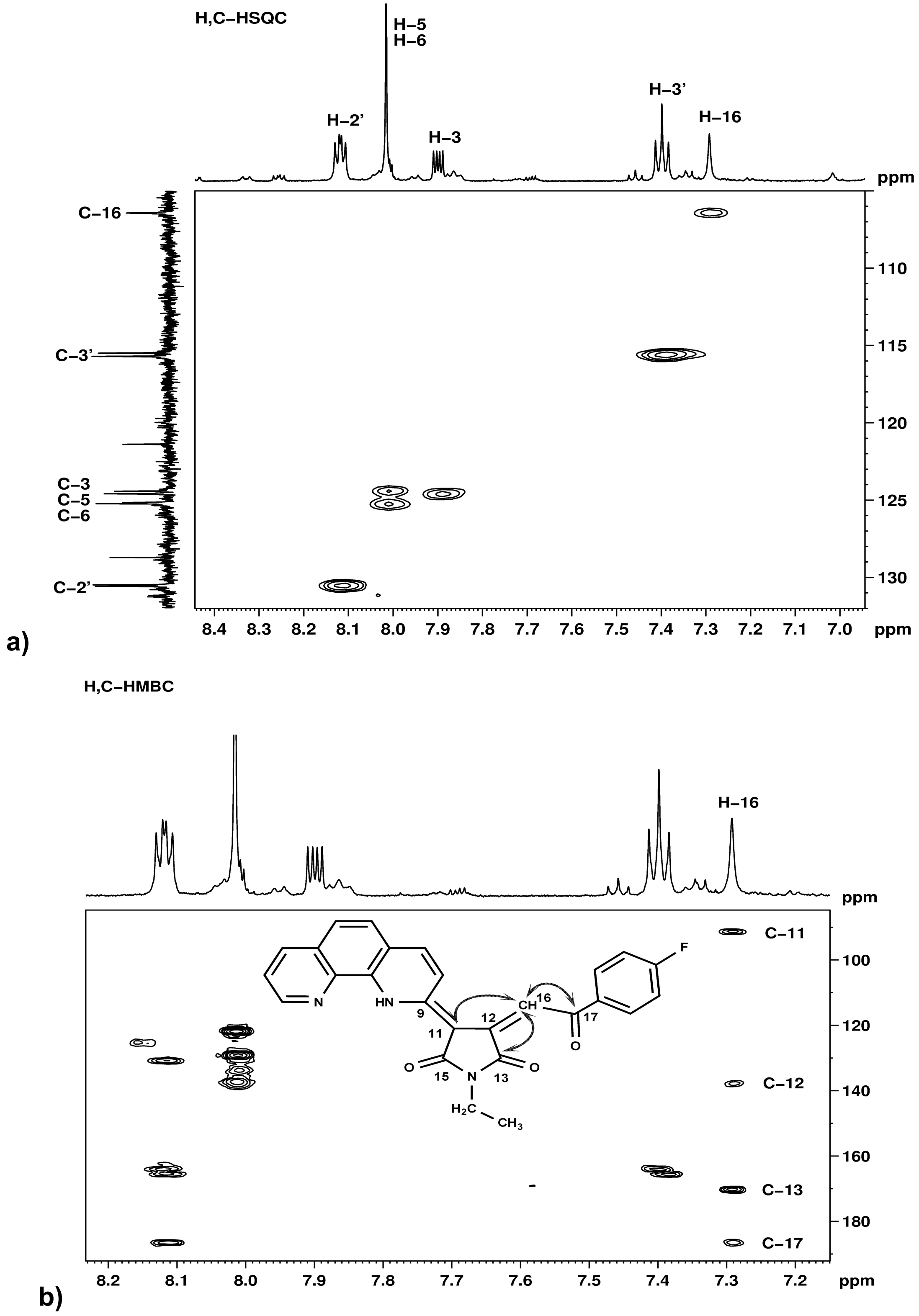

Figure S14. **a)** Detailed low-field region of the H,C-HSQC spectrum showing the correlation signals for direct proton-carbon bonds. **b)** Detailed low-field region of the H,C-HMBC spectrum showing proton-carbon long-range correlations that support the proposed chemical structure for **6a**.

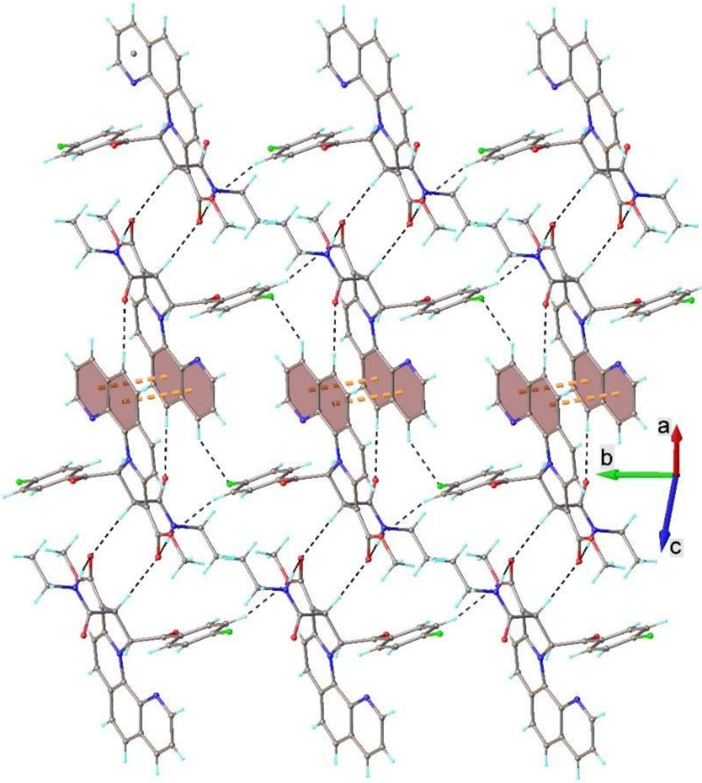

Figure S15. View of 2D supramolecular layer in the crystal structure of **4a** showing the role of hydrogen bonding and stacking interactions. Centroid-to-centroid distances of 3.575(5) Å are drawn as black-dashed lines.
H-bonds parameters: C3-H···F1 [C3-H 0.93 Å, H···F1 2.53 Å, C3···F1(2 −*x*, 2 −*y*, −*z*) 3.329(3) Å, ∠C3HF1 144.6°; C5-H···O4 [C3-H 0.93 Å, H···O4 2.42 Å, C5···O4(2 −*x*, 1 −*y*, −*z*) 3.342(3) Å, ∠C5HO4 170.9°; C9-H···O1 [C3-H 0.93 Å, H···O1 2.33 Å, C9···O1(2 −*x*, 2 −*y*, −*z*) 3.329(3) Å, ∠C9HO1 144.6°; C17-H···O1 [C3-H 0.93 Å, H···O1 2.56 Å, C17···O1(*x*, 1 + *y*, *z*) 2.875(3) Å, ∠C17HO1 116.9°; C22-H···O2 [C3-H 0.98 Å, H···O2 2.51 Å, C22···O2(1 −*x*, 1 −*y*, 1 −*z*) 3.462(2) Å, ∠C22HO2 165.3°.

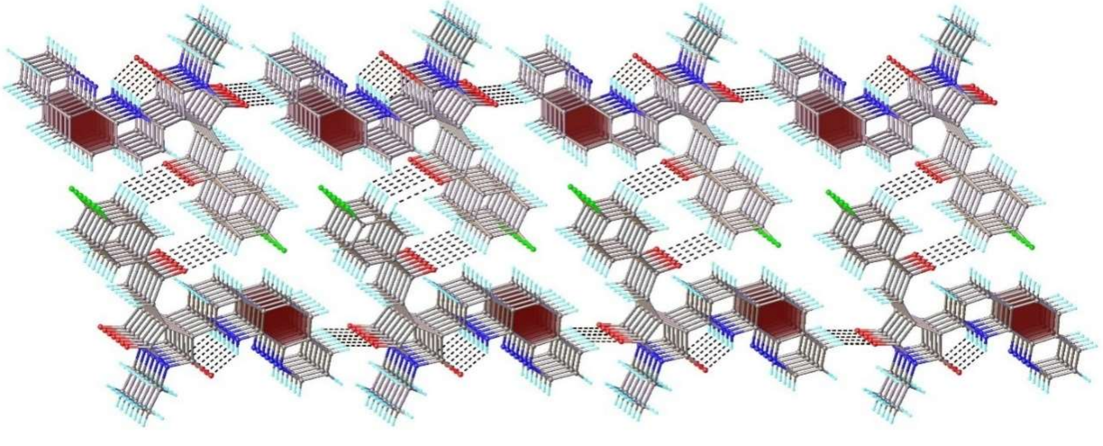

Figure S16. 3D supramolecular network in the crystal of **5a**. Aromatic rings involved in π-π stacking are shown in brown color. Centroid-to-centroid distance is of 3.6744(2) Å.
H-bonds parameters: C3-H···O2 [C3-H 0.95 Å, H···O2 2.39 Å, C3···O2(*x* – 1, *y* – 1, *z*) 3.343(3) Å, ∠C3HO2 149.7°; C21-H···O3 [C3-H 0.95 Å, H···O3 2.60 Å, C21···O3(1 −*x*, *y* – 0.5,0.5 – *z*) 3.280(3) Å, ∠C21HO3 129.1°

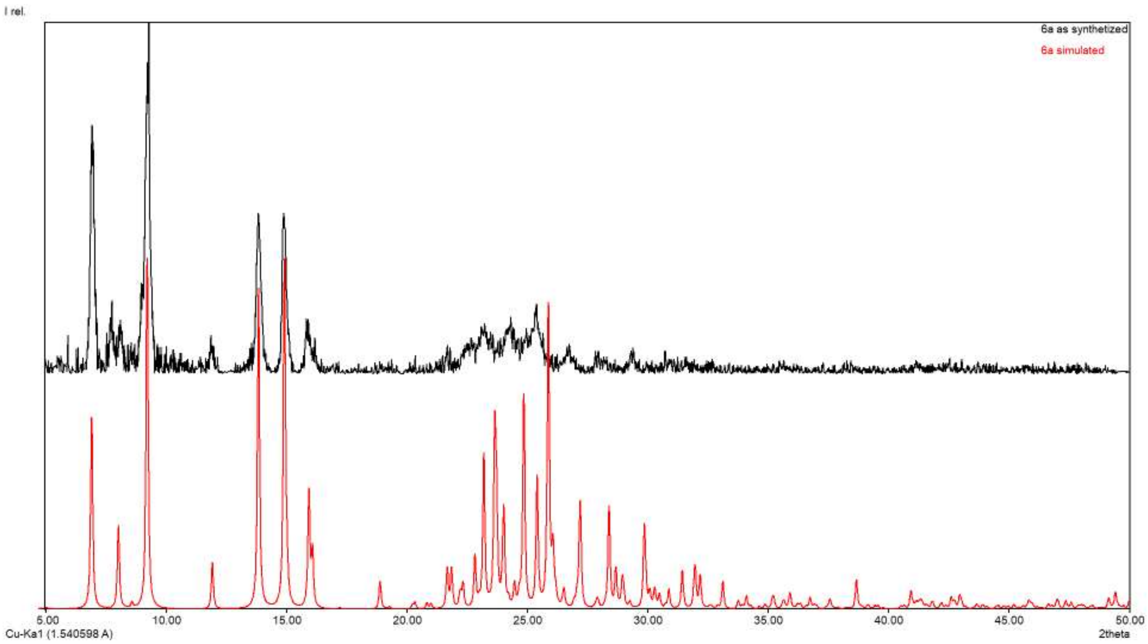

Figure S17. The PXRD patterns of the derivative **5a** showing the similitude between simulated and experimental diffraction peaks.

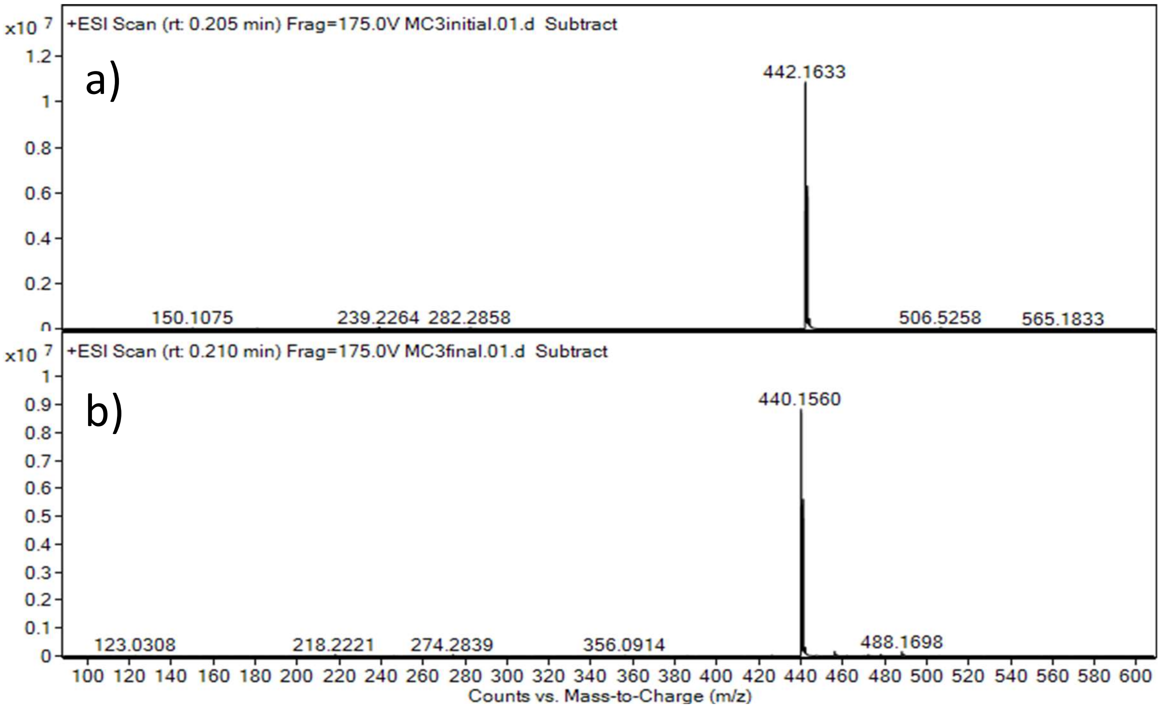

Figure S18. Positive ESI mass spectra associated with compounds **2a** (a) and **6a** (b).

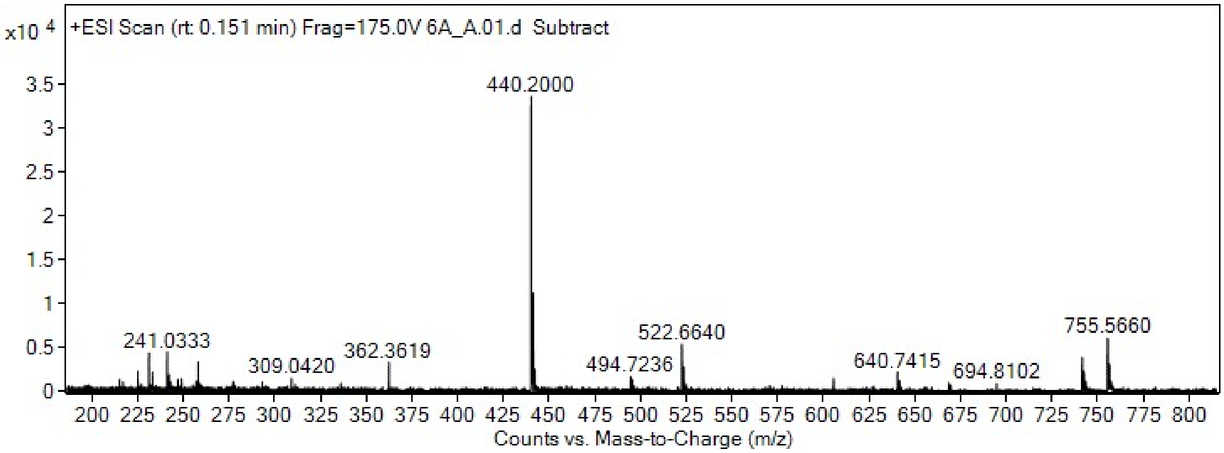

Figure S19. Positive ESI mass spectrum associated with compound **6a** in acidic conditions.

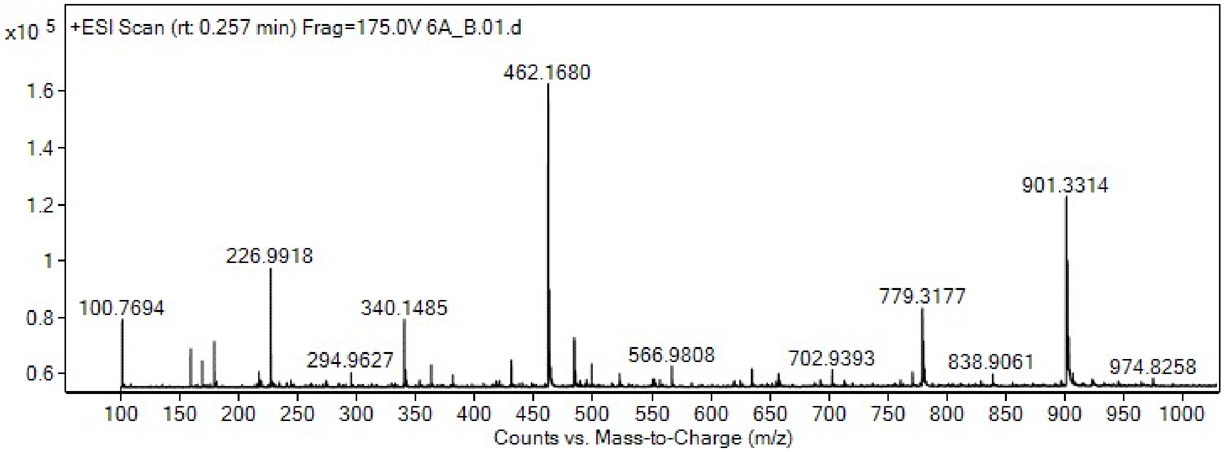

Figure S20. Positive ESI mass spectrum associated with compound **6a** in basic conditions.

Table S1. Bond distances (Å) and angles (°).

Compound **2a**.

F1-C18	1.360(2)	N2-C13	1.484(2)	C7-C8	1.428(3)	C15-C20	1.389(3)		
O1-C23	1.353(2)	N3-C25	1.332(3)	C7-C11	1.392(3)	C16-C17	1.385(3)		
O1-C24	1.434(2)	N3-C26	1.453(3)	C8-C9	1.338(3)	C17-C18	1.359(3)		
O2-C23	1.221(2)	C1-C2	1.394(3)	C9-C10	1.429(3)	C18-C19	1.351(3)		
O3-C14	1.216(2)	C2-C3	1.347(3)	C10-C21	1.375(3)	C19-C20	1.381(3)		
O4-C25	1.219(2)	C3-C4	1.402(3)	C11-C12	1.439(3)	C21-C22	1.514(2)		
N1-C1	1.313(3)	C4-C5	1.416(3)	C13-C14	1.518(3)	C21-C23	1.420(3)		
N1-C12	1.354(2)	C4-C12	1.415(3)	C13-C22	1.539(3)	C22-C25	1.537(3)		
N2-C10	1.383(2)	C5-C6	1.350(3)	C14-C15	1.486(3)	C26-C27	1.494(3)		
N2-C11	1.380(2)	C6-C7	1.414(3)	C15-C16	1.389(3)				

C23-O1-C24	116.70(17)	N2-C10-C9	117.30(17)	C18-C17-C16	118.5(2)
C1-N1-C12	118.28(18)	C21-C10-N2	111.30(16)	C17-C18-F1	117.9(2)

C10-N2-C13	110.08(15)	C21-C10-C9	131.40(18)	C19-C18-F1	118.9(3)		
C11-N2-C10	123.81(16)	N2-C11-C7	118.09(17)	C19-C18-C17	123.2(2)		
C11-N2-C13	126.11(15)	N2-C11-C12	123.09(17)	C18-C19-C20	118.5(2)		
C25-N3-C26	123.60(18)	C7-C11-C12	118.82(18)	C19-C20-C15	120.8(2)		
N1-C1-C2	123.9(2)	N1-C12-C4	121.77(18)	C10-C21-C22	109.09(16)		
C3-C2-C1	118.4(2)	N1-C12-C11	119.50(17)	C10-C21-C23	128.77(17)		
C2-C3-C4	120.6(2)	C4-C12-C11	118.72(18)	C23-C21-C22	122.14(16)		
C3-C4-C5	122.3(2)	N2-C13-C14	110.18(16)	C21-C22-C13	103.44(15)		
C3-C4-C12	117.1(2)	N2-C13-C22	103.93(14)	C21-C22-C25	111.05(17)		
C12-C4-C5	120.65(19)	C14-C13-C22	111.53(15)	C25-C22-C13	111.34(15)		
C6-C5-C4	119.26(19)	O3-C14-C13	120.11(18)	O1-C23-C21	114.01(17)		
C5-C6-C7	122.1(2)	O3-C14-C15	121.4(2)	O2-C23-O1	121.08(19)		
C6-C7-C8	121.52(19)	C15-C14-C13	118.34(18)	O2-C23-C21	124.91(19)		
C11-C7-C6	120.06(19)	C16-C15-C14	118.32(19)	O4-C25-N3	122.4(2)		
C11-C7-C8	118.42(18)	C16-C15-C20	118.5(2)	O4-C25-C22	122.78(19)		
C9-C8-C7	122.43(18)	C20-C15-C14	123.2(2)	N3-C25-C22	114.82(17)		
C8-C9-C10	119.44(18)	C17-C16-C15	120.5(2)	N3-C26-C27	111.3(2)		

Compound **4a**.

F1-C18	1.360(2)	C1-C2	1.394(3)	C13-C14	1.518(3)		
O1-C23	1.353(2)	C2-C3	1.347(3)	C13-C22	1.539(3)		
O1-C24	1.434(2)	C3-C4	1.402(3)	C14-C15	1.486(3)		
O2-C23	1.221(2)	C4-C5	1.416(3)	C15-C16	1.389(3)		
O3-C14	1.216(2)	C4-C12	1.415(3)	C15-C20	1.389(3)		
O4-C25	1.219(2)	C5-C6	1.350(3)	C16-C17	1.385(3)		
N1-C1	1.313(3)	C6-C7	1.414(3)	C17-C18	1.359(3)		
N1-C12	1.354(2)	C7-C8	1.428(3)	C18-C19	1.351(3)		
N2-C10	1.383(2)	C7-C11	1.392(3)	C19-C20	1.381(3)		
N2-C11	1.380(2)	C8-C9	1.338(3)	C21-C22	1.514(2)		
N2-C13	1.484(2)	C9-C10	1.429(3)	C21-C23	1.420(3)		
N3-C25	1.332(3)	C10-C21	1.375(3)	C22-C25	1.537(3)		
N3-C26	1.453(3)	C11-C12	1.439(3)	C26-C27	1.494(3)		

C23-O1-C24	116.70(17)	N2-C10-C9	117.30(17)	C18-C17-C16	118.5(2)		
C1-N1-C12	118.28(18)	C21-C10-N2	111.30(16)	C17-C18-F1	117.9(2)		
C10-N2-C13	110.08(15)	C21-C10-C9	131.40(18)	C19-C18-F1	118.9(3)		
C11-N2-C10	123.81(16)	N2-C11-C7	118.09(17)	C19-C18-C17	123.2(2)		
C11-N2-C13	126.11(15)	N2-C11-C12	123.09(17)	C18-C19-C20	118.5(2)		
C25-N3-C26	123.60(18)	C7-C11-C12	118.82(18)	C19-C20-C15	120.8(2)		
N1-C1-C2	123.9(2)	N1-C12-C4	121.77(18)	C10-C21-C22	109.09(16)		
C3-C2-C1	118.4(2)	N1-C12-C11	119.50(17)	C10-C21-C23	128.77(17)		
C2-C3-C4	120.6(2)	C4-C12-C11	118.72(18)	C23-C21-C22	122.14(16)		
C3-C4-C5	122.3(2)	N2-C13-C14	110.18(16)	C21-C22-C13	103.44(15)		
C3-C4-C12	117.1(2)	N2-C13-C22	103.93(14)	C21-C22-C25	111.05(17)		
C12-C4-C5	120.65(19)	C14-C13-C22	111.53(15)	C25-C22-C13	111.34(15)		
C6-C5-C4	119.26(19)	O3-C14-C13	120.11(18)	O1-C23-C21	114.01(17)		

C5-C6-C7	122.1(2)	O3-C14-C15	121.4(2)	O2-C23-O1	121.08(19)
C6-C7-C8	121.52(19)	C15-C14-C13	118.34(18)	O2-C23-C21	124.91(19)
C11-C7-C6	120.06(19)	C16-C15-C14	118.32(19)	O4-C25-N3	122.4(2)
C11-C7-C8	118.42(18)	C16-C15-C20	118.5(2)	O4-C25-C22	122.78(19)
C9-C8-C7	122.43(18)	C20-C15-C14	123.2(2)	N3-C25-C22	114.82(17)
C8-C9-C10	119.44(18)	C17-C16-C15	120.5(2)	N3-C26-C27	111.3(2)

Compound **6a**.

F1-C22	1.374(3)	C3-C4	1.415(4)	C13-C16	1.430(3)		
O1-C14	1.228(3)	C4-C5	1.432(4)	C15-C16	1.512(3)		
O2-C15	1.216(3)	C4-C9	1.409(4)	C16-C17	1.367(3)		
O3-C18	1.235(3)	C5-C6	1.347(4)	C17-C18	1.451(3)		
N1-C1	1.321(3)	C6-C7	1.429(3)	C18-C19	1.505(4)		
N1-C9	1.359(3)	C7-C8	1.401(4)	C19-C20	1.385(3)		
N2-C8	1.364(3)	C7-C10	1.410(4)	C19-C24	1.382(4)		
N2-C12	1.362(3)	C8-C9	1.425(4)	C20-C21	1.374(4)		
N3-C14	1.397(3)	C10-C11	1.360(3)	C21-C22	1.352(4)		
N3-C15	1.375(3)	C11-C12	1.423(3)	C22-C23	1.375(4)		
N3-C25	1.460(3)	C12-C13	1.409(3)	C23-C24	1.397(4)		
C1-C2	1.392(4)	C13-C14	1.457(3)	C25-C26	1.507(3)		
C2-C3	1.359(4)						

C1-N1-C9	116.7(2)	C7-C8-C9	121.7(2)	C13-C16-C15	106.0(2)
C12-N2-C8	125.2(2)	N1-C9-C4	123.8(3)	C17-C16-C13	139.5(2)
C14-N3-C25	124.6(2)	N1-C9-C8	117.6(2)	C17-C16-C15	114.3(2)
C15-N3-C14	110.6(2)	C4-C9-C8	118.6(3)	C16-C17-C18	129.9(3)
C15-N3-C25	124.6(2)	C11-C10-C7	122.3(2)	O3-C18-C17	121.9(3)
N1-C1-C2	123.9(3)	C10-C11-C12	120.6(3)	O3-C18-C19	119.6(2)
C3-C2-C1	120.0(3)	N2-C12-C11	115.5(2)	C17-C18-C19	118.5(2)
C2-C3-C4	118.8(3)	N2-C12-C13	117.7(2)	C20-C19-C18	118.8(2)
C3-C4-C5	124.0(3)	C13-C12-C11	126.8(2)	C24-C19-C18	122.9(2)
C9-C4-C3	116.9(3)	C12-C13-C14	120.4(2)	C24-C19-C20	118.3(3)
C9-C4-C5	119.0(3)	C12-C13-C16	132.5(2)	C21-C20-C19	121.4(3)
C6-C5-C4	121.4(3)	C16-C13-C14	107.1(2)	C22-C21-C20	118.2(3)
C5-C6-C7	121.3(3)	O1-C14-N3	122.0(2)	F1-C22-C23	117.6(3)
C8-C7-C6	117.9(3)	O1-C14-C13	129.3(2)	C21-C22-F1	118.5(3)
C8-C7-C10	116.7(2)	N3-C14-C13	108.7(2)	C21-C22-C23	123.9(3)
C10-C7-C6	125.4(3)	O2-C15-N3	124.7(2)	C22-C23-C24	116.6(3)
N2-C8-C7	119.4(3)	O2-C15-C16	127.9(2)	C19-C24-C23	121.5(3)
N2-C8-C9	118.9(2)	N3-C15-C16	107.3(2)	N3-C25-C26	113.16(18)

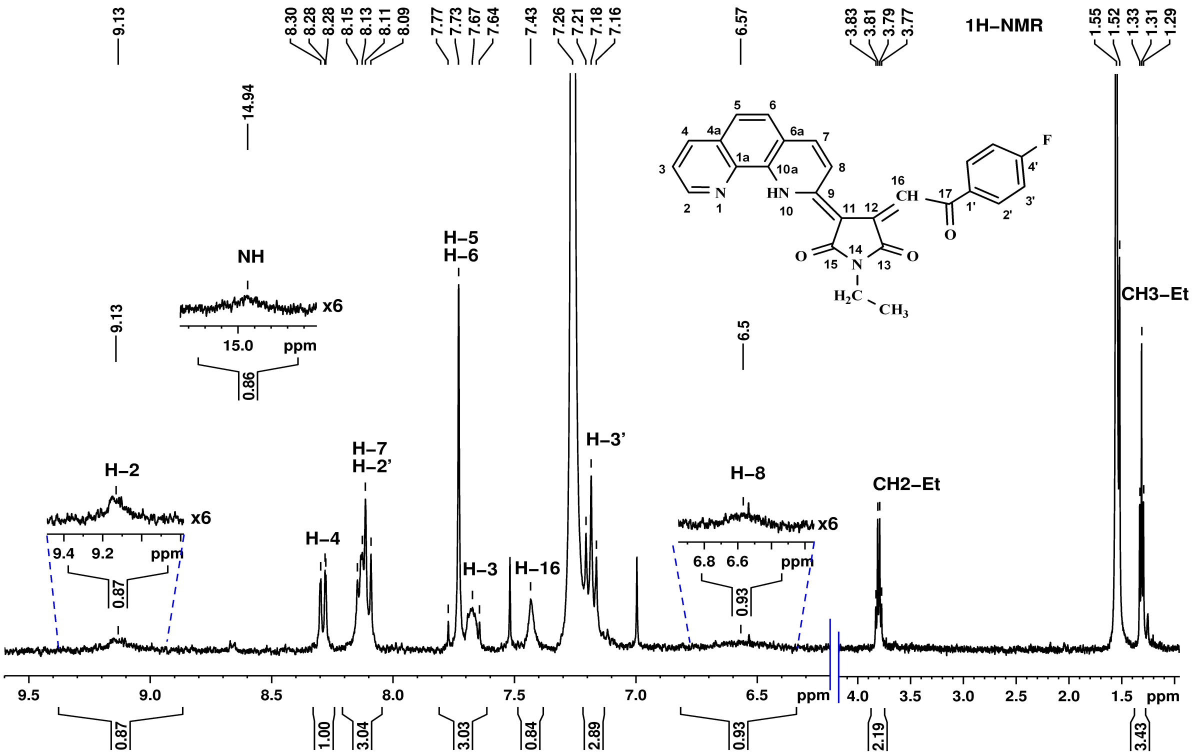

Figure S21. The ^{1}H-NMR spectrum corresponding to compound **6a**, recorded in $CDCl_3$ at room temperature, on a 400 MHz spectrometer.

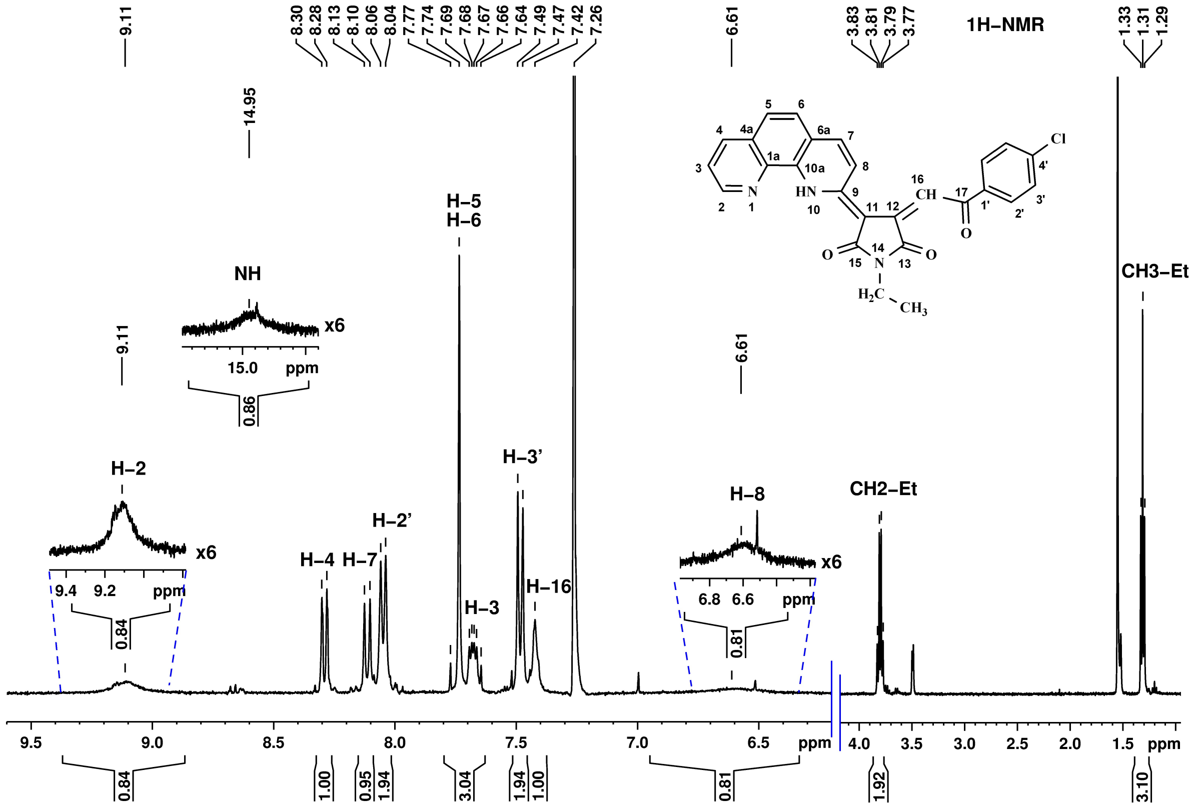

Figure S22. The ^{1}H-NMR spectrum corresponding to compound **6b**, recorded in $CDCl_3$ at room temperature, on a 400 MHz spectrometer. At room temperature, the signals from 6.60 ppm (H-8), 9.10 ppm (H-2) and 14.92 ppm (NH) are very broad and close to the baseline.

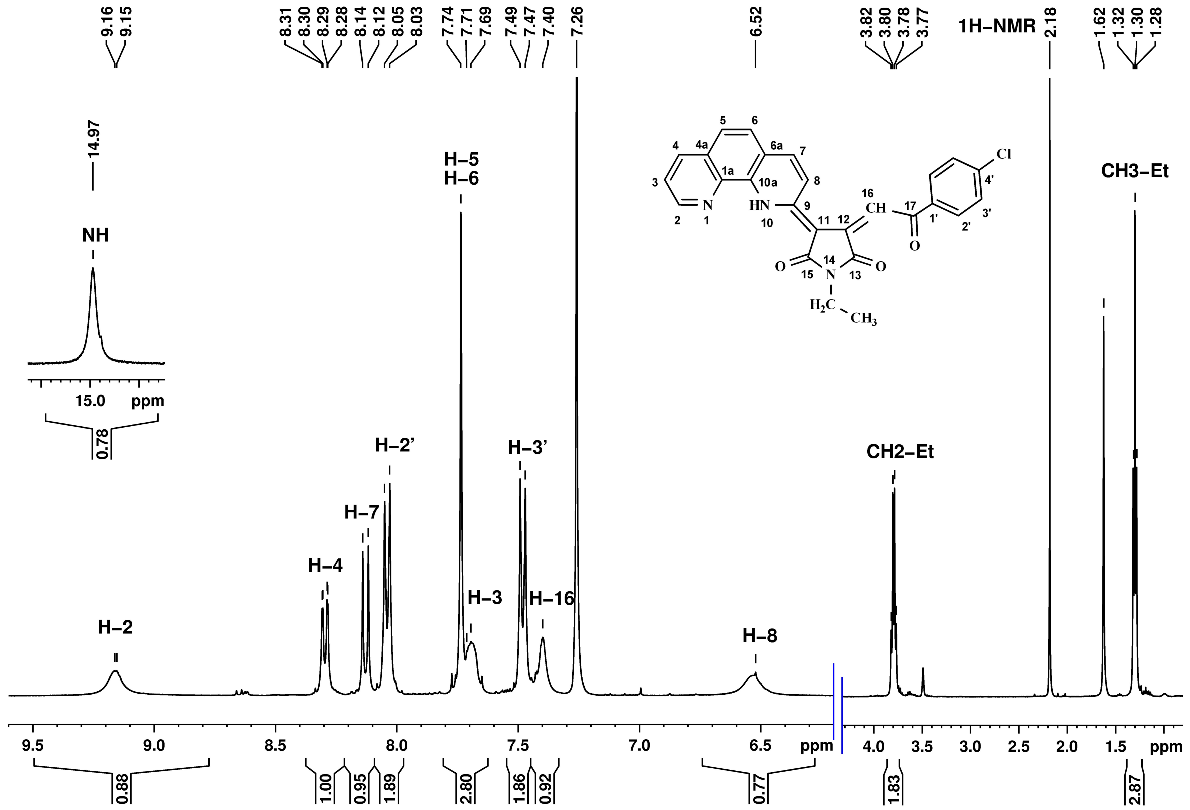

Figure S23. The ^{1}H-NMR spectrum corresponding to compound **6b**, recorded in $CDCl_3$ at 5 °C, on a 400 MHz spectrometer. At this lower temperature, the broad signals from 6.5 ppm (H-8), 9.15 ppm (H-2) and 14.97 ppm (NH) became sharper, being more visible from the baseline.

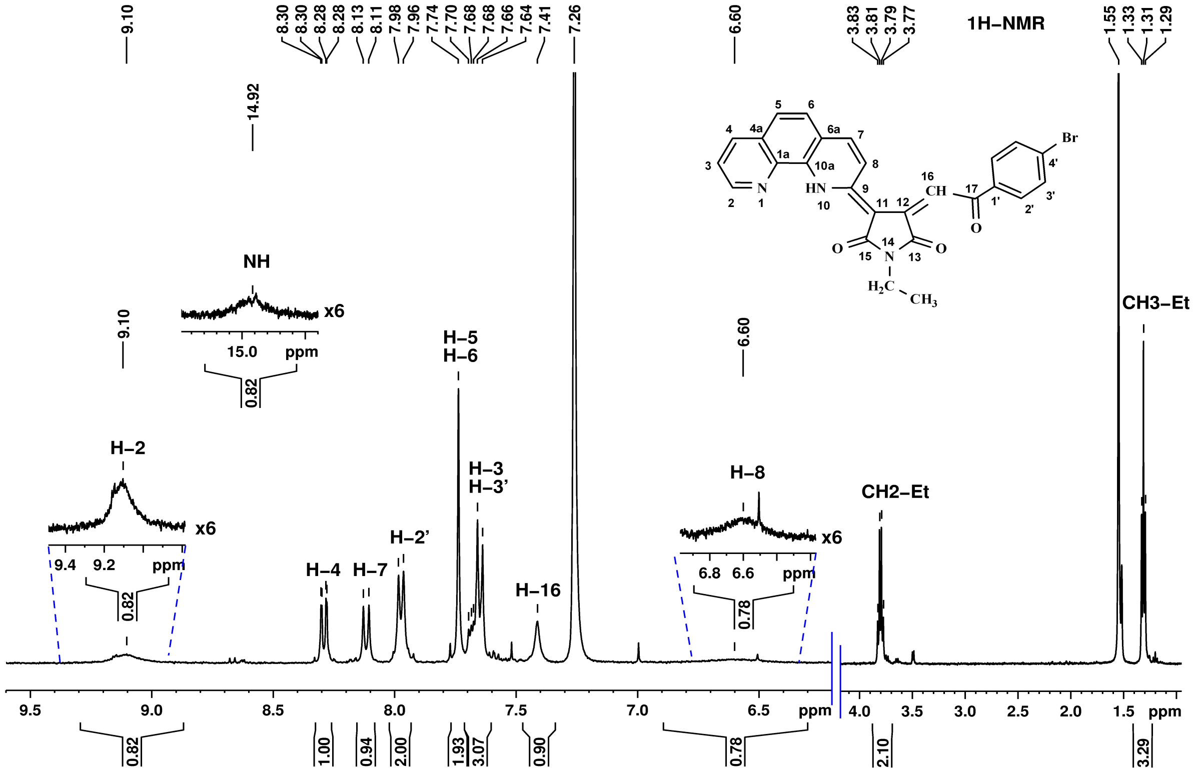

Figure S24. The ^{1}H-NMR spectrum corresponding to compound **6c**, recorded in $CDCl_3$ at room temperature, on a 400 MHz spectrometer.

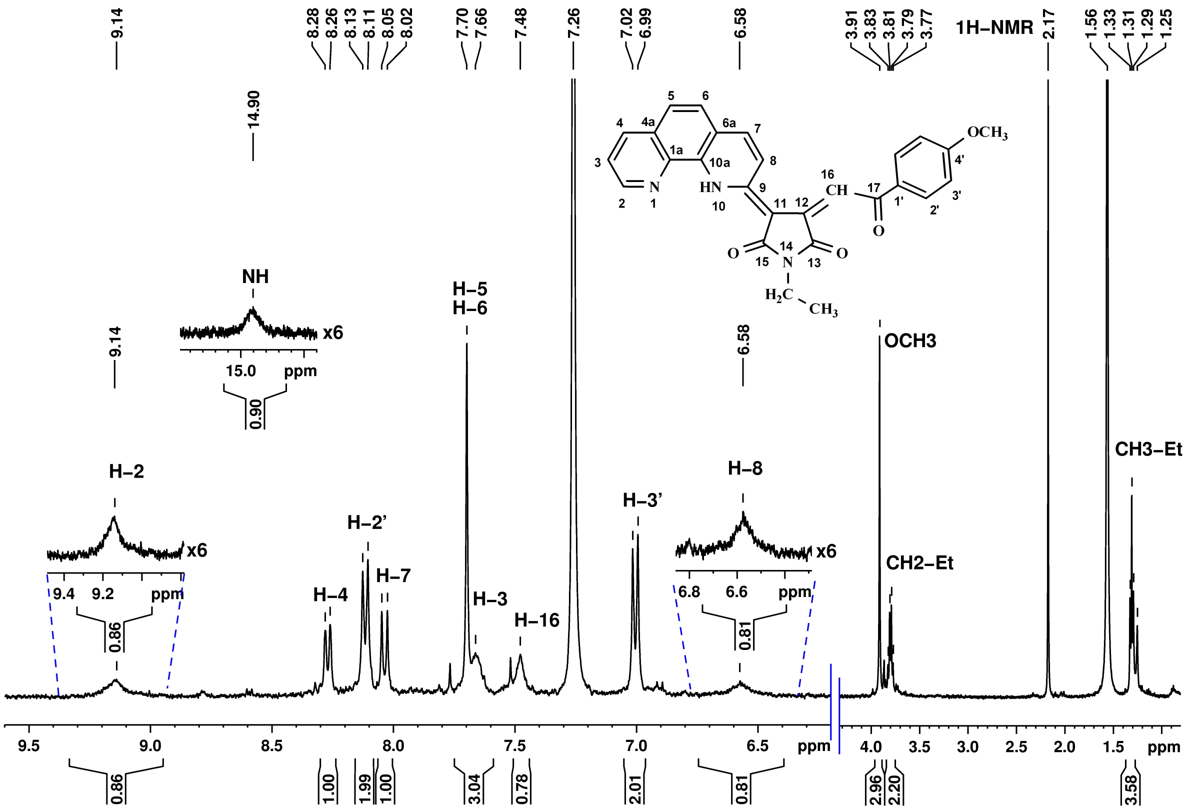

Figure S25. The ^{1}H-NMR spectrum corresponding to compound **6d**, recorded in $CDCl_3$ at room temperature, on a 400 MHz spectrometer.
